# Supplementary material for: Greater nurse density correlates to higher level of population ageing globally, but is more prominent in developed countries
Source: PLoS One. 2023 Sep 29;18(9):e0292371. doi: 10.1371/journal.pone.0292371 (PMC10540962; doi:10.1371/journal.pone.0292371)
Supplement: Attachment — Submitted filename: PONE-D-22-35617_R3_reviewer1.pdf [file pone.0292371.s007.pdf]

PLOS ONE

Greater nurse density correlates to higher level of population ageing globally, but is more prominent in developed countries.

--Manuscript Draft--

**Manuscript Number:**	PONE-D-22-35617R3
**Article Type:**	Research Article
**Full Title:**	Greater nurse density correlates to higher level of population ageing globally, but is more prominent in developed countries.
**Short Title:**	Nurses promote population ageing
**Corresponding Author:**	Wenpeng You<br>The University of Adelaide - North Terrace Campus: The University of Adelaide<br>Adelaide, AUSTRALIA
**Keywords:**	ursing healthcare, Population ageing, Nursing staff shortage, Nursing workforce, Nurse density
**Abstract:**	Background<br>Representing over 50% of the healthcare workforce, nurses provide care to people at all ages. This study advances, at a population level, that high levels of nursing services, measured by nurse density may significantly promote population ageing measured by the percentage of a population over 65 years of age (65yo%).<br>Methods<br>Population level data was examined to explore the correlation between nurse density and 65yo%. The confounding impacts on ageing such as the effects of economic affluence, physician density, fertility rate, obesity and urban advantages were also considered. Scatter plots, bivariate correlation, partial correlation and multiple linear regression analyses were performed for examining the correlations.<br>Results<br>Nurse density correlated to 65yo%; this relationship was independent of other influences such as fertility rate, economic affluence, obesity prevalence, physician density and urban advantages. Second to fertility rate, nursing density had the greatest influence on 65yo%. The predicting and confounding variables explain 74.4% of the total 65yo% variance. The universal correlations identified in country groupings suggest that low nurse density may be a significant global concern.<br>Conclusions<br>While nurse density might contribute significantly to 65yo% globally, the effect was more prominent in developed countries. Ironically, countries with higher nurse densities and therefore greater levels of 65yo%, were countries with an increased need for more nursing staff. To highlight the profound implications for the role the nursing profession plays especially at a time of global nursing shortage, further study into the effects of long-run elasticity of nurse staffing level on population ageing may be needed. For instance, what percentage of increase of nursing staff would be required to meet every 1% increase of an ageing population.
**Order of Authors:**	Wenpeng You
	Frank Donnelly
**Response to Reviewers:**	Please see attached document titled, 0 Response to Review Decision
**Additional Information:**	
**Question**	**Response**
**Financial Disclosure**<br><br>Enter a financial disclosure statement that describes the sources of funding for the work included in this submission. Review the submission guidelines for detailed	The author(s) received no specific funding for this work.

Powered by Editorial Manager® and ProduXion Manager® from Aries Systems Corporation

requirements. View published research articles from *PLOS ONE* for specific examples.<br><br>This statement is required for submission and **will appear in the published article** if the submission is accepted. Please make sure it is accurate.<br><br>**Unfunded studies**<br>Enter: *The author(s) received no specific funding for this work.*<br><br>**Funded studies**<br>Enter a statement with the following details:<br>• Initials of the authors who received each award<br>• Grant numbers awarded to each author<br>• The full name of each funder<br>• URL of each funder website<br>• Did the sponsors or funders play any role in the study design, data collection and analysis, decision to publish, or preparation of the manuscript?<br>• **NO** - Include this sentence at the end of your statement: *The funders had no role in study design, data collection and analysis, decision to publish, or preparation of the manuscript.*<br>• **YES** - Specify the role(s) played.<br><br>* typeset	
**Competing Interests**<br><br>Use the instructions below to enter a competing interest statement for this submission. On behalf of all authors, disclose any competing interests that could be perceived to bias this work—acknowledging all financial support and any other relevant financial or non-financial competing interests.<br><br>This statement is required for submission and **will appear in the published article** if the submission is accepted. Please make sure it is accurate and that any funding sources listed in your Funding Information later in the submission form are also declared in your Financial Disclosure	The authors have declared that no competing interests exist.

statement.<br><br>View published research articles from *PLOS ONE* for specific examples.<br><br>**NO authors have competing interests**<br><br>Enter: *The authors have declared that no competing interests exist.*<br><br>**Authors with competing interests**<br><br>Enter competing interest details beginning with this statement:<br><br>*I have read the journal's policy and the authors of this manuscript have the following competing interests: [insert competing interests here]*<br><br>* typeset	
**Ethics Statement**<br><br>Enter an ethics statement for this submission. This statement is required if the study involved:<br><br>• Human participants<br>• Human specimens or tissue<br>• Vertebrate animals or cephalopods<br>• Vertebrate embryos or tissues<br>• Field research<br><br>Write "N/A" if the submission does not require an ethics statement.<br><br>General guidance is provided below. Consult the submission guidelines for detailed instructions. **Make sure that all information entered here is included in the Methods section of the manuscript.**	The relevant United Nations agencies offer free online access to data required for the analyses in this study. The data can only be identifiable to the level of population. They are not identifiable or re-identifiable to the individual participant, their family and community. Therefore, there is no need to obtain the ethical approval or consent during our entire study process.

Powered by Editorial Manager® and ProduXion Manager® from Aries Systems Corporation

**Format for specific study types**<br><br>**Human Subject Research (involving human participants and/or tissue)**<br>• Give the name of the institutional review board or ethics committee that approved the study<br>• Include the approval number and/or a statement indicating approval of this research<br>• Indicate the form of consent obtained (written/oral) or the reason that consent was not obtained (e.g. the data were analyzed anonymously)<br><br>**Animal Research (involving vertebrate animals, embryos or tissues)**<br>• Provide the name of the Institutional Animal Care and Use Committee (IACUC) or other relevant ethics board that reviewed the study protocol, and indicate whether they approved this research or granted a formal waiver of ethical approval<br>• Include an approval number if one was obtained<br>• If the study involved *non-human primates*, add *additional details* about animal welfare and steps taken to ameliorate suffering<br>• If anesthesia, euthanasia, or any kind of animal sacrifice is part of the study, include briefly which substances and/or methods were applied<br><br>**Field Research**<br><br>Include the following details if this study involves the collection of plant, animal, or other materials from a natural setting:<br>• Field permit number<br>• Name of the institution or relevant body that granted permission	
**Data Availability**<br><br>Authors are required to make all data underlying the findings described fully available, without restriction, and from the time of publication. PLOS allows rare exceptions to address legal and ethical concerns. See the PLOS Data Policy and FAQ for detailed information.	Yes - all data are fully available without restriction

A Data Availability Statement describing where the data can be found is required at submission. Your answers to this question constitute the Data Availability Statement and **will be published in the article**, if accepted.<br><br>**Important:** Stating 'data available on request from the author' is not sufficient. If your data are only available upon request, select 'No' for the first question and explain your exceptional situation in the text box.<br><br>Do the authors confirm that all data underlying the findings described in their manuscript are fully available without restriction?	
**Describe where the data may be found in full sentences. If you are copying our sample text, replace any instances of XXX with the appropriate details.**<br><br>• If the data are **held or will be held in a public repository**, include URLs, accession numbers or DOIs. If this information will only be available after acceptance, indicate this by ticking the box below. For example: *All XXX files are available from the XXX database (accession number(s) XXX, XXX.).*<br>• If the data are all contained **within the manuscript and/or Supporting Information files**, enter the following: *All relevant data are within the manuscript and its Supporting Information files.*<br>• If neither of these applies but you are able to provide **details of access elsewhere**, with or without limitations, please do so. For example:<br><br>*Data cannot be shared publicly because of [XXX]. Data are available from the XXX Institutional Data Access / Ethics Committee (contact via XXX) for researchers who meet the criteria for access to confidential data.*<br><br>*The data underlying the results presented in the study are available from (include the name of the third party*	All relevant data sources are described within the manuscript.

and contact information or URL).<br>• This text is appropriate if the data are owned by a third party and authors do not have permission to share the data.<br><br>* typeset	
Additional data availability information:	

Manuscript

Click here to access/download;Manuscript;1 ms Nurs n ageing 27062023 Clean.docx

Greater nurse density correlates to higher level of population ageing globally, but is more prominent in developed countries.

Wenpeng You, PhD[1,2,3], Frank Donnelly, PhD[1]

[1] Adelaide Nursing School, the University of Adelaide, Adelaide, Australia

[2] Heart and Lung, Royal Adelaide Hospital, Adelaide, Australia

[3] Adelaide Medical School, the University of Adelaide, Adelaide, Australia

Running Head: Nurses promote population ageing

Corresponding author:

Wenpeng You, PhD, RN

wenpeng.you@adelaide.edu.au

Abstract

Background

Representing over 50% of the healthcare workforce, nurses provide care to people at all ages. This study advances, at a population level, that high levels of nursing services, measured by nurse density may significantly promote population ageing measured by the percentage of a population over 65 years of age (65yo%).

Methods

Population level data was examined to explore the correlation between nurse density and 65yo%. The confounding impacts on ageing such as the effects of economic affluence, physician density, fertility rate, obesity and urban advantages were also considered. Scatter plots, bivariate correlation, partial correlation and multiple linear regression analyses were performed for examining the correlations.

Results

Nurse density correlated to 65yo%; this relationship was independent of other influences such as fertility rate, economic affluence, obesity prevalence, physician density and urban advantages. Second to fertility rate, nursing density had the greatest influence on 65yo%. The predicting and confounding variables explain 74.4% of the total 65yo% variance. The universal correlations identified in country groupings suggest that low nurse density may be a significant global concern.

Conclusions

While nurse density might contribute significantly to 65yo% globally, the effect was more prominent in developed countries. Ironically, countries with higher nurse densities and therefore greater levels of 65yo%, were countries with an increased need for more nursing staff. To highlight the profound implications for the

role the nursing profession plays especially at a time of global nursing shortage, further study into the effects of long-run elasticity of nurse staffing level on population ageing may be needed. For instance, what percentage of increase of nursing staff would be required to meet every 1% increase of an ageing population.

Keywords:

Nursing healthcare, Population ageing, Nursing staff shortage, Nursing workforce, Nurse density

Introduction

Over the last 200 years, people around the world have achieved impressive progress in health represented by an increase of the number of people aged 65 and over. Population ageing refers to changes in the age composition of a population which increases the proportion of older persons. It has become the 21st century's dominant demographic measure. Demographically, the most often used index of population ageing is the percentage of population segment aged 65 and over (65yo% hereafter) [1, 2].

It is well-established that declining fertility rates and rising life expectancy are the primary causes for population ageing [3-6]. Without sustainable numbers of younger people to replace the ageing population, the relative size of the population segment aged 65 years old or over will continue to increase. Improved economic circumstances have been exacerbating this impact because of increased welfare and life satisfaction [7]. Other studies identify factors, such as education [8] and genetic traits [9] that may also be contributing factors impacting population ageing.

The evolution of the nursing workforce into an independent health care profession is evident as nurses now account for over 50% of the global healthcare industry [10]. Worldwide, nurses have and will continue to play a critical role in health promotion, disease prevention and delivery of a comprehensive range of healthcare services [10]. Within primary, secondary and tertiary healthcare settings, the scope of nursing healthcare has afforded nurses' opportunities to lead and coordinate patient care provided by multidisciplinary health professionals. Therefore, the quality of healthcare from nurses should be considered a core indicator of a populations level of healthcare. Previous studies, for example, have revealed that nursing workforce plays a significant role in reducing infant and neonatal mortalities [11] and life expectancy at birth and at 65 years old [12, 13]. An outcome from the role of nursing workforce in promoting population health has been an increase in the number of who survive past 65 years old.

Ironically, as the population of many countries pushing past 65yo increase, these countries have the burdens of an ageing population started to further impact available healthcare systems. In recent years the level of demand has grown, at a much faster pace [14]. This impact has contributed to the global nursing staff shortage crisis because old people have higher healthcare dependency on healthcare professionals, especially nursing staff. Studies in individual healthcare facility settings or at community levels have reported that nursing shortages are a chronic issue [15], and this was highlighted during the COVID-19 pandemic period [16].

The challenge for health authorities is to take strategic actions to provide sufficient healthcare to an older population with high demand of nursing care. This study helps to illustrate and quantify the role of nursing

workforce in increasing the proportion of people over 65 years old. In this study, we hypothesized that, globally, nursing healthcare services may be a significant contributor for increasing the portion of people to live over 65 years. We tested this hypothesis by examining the statistical relationship between nurse density and the 65yo%, globally and regionally. With reference to previous studies into healthcare outcomes of nursing staff, economic affluence, obesity prevalence, physician density, total fertility rate and urbanization were incorporated as potential confounding factors.

Materials and Methods

Data Sources

The country specific data published by the agencies of the United Nations (UN) were extracted for this ecological study. Country in this study does not necessarily indicate political independence but only refers to the geographic territory or region which reported data on health, demography and economic situation to the World Bank [17]. Except for the obesity prevalence rate which was extracted from the World Health Organization [18], all other variables (nurse density, population ageing level, GDP PPP, physician density, total fertility rate and urbanization) were downloaded from the World Bank database [19].

1. The dependent variable, population ageing level indexed with the percentage of population aged 65 and above (65yo%) in 2020 [20]

The DataBank of the World Bank is an analysis and visualisation tool that contains collections of time series data on a variety of topics, including socioeconomics, key health, nutrition and demography statistics. Population is based on the de facto definition of population, which counts all residents regardless of legal residency status or citizenship.

2. The independent variable, nurse density measured with the number of nurses and midwives per 1,000 people [21]

To reduce random errors at the time of data collection, we averaged the number of nurses and midwives for the period between 2014 and 2018 in each country for our data analyses.

Empirically and as per recent studies, economic affluence, obesity, physician service access, total fertility rate and urban living have been either positive or negatively associated with people's health affecting their opportunities surviving 65 years old. Therefore, they were included as the potential confounders while we analysed the independent role of nursing workforce in promoting population ageing:

3. Economic affluence, expressed with the gross domestic product (GDP) per capita [22]

The economic affluence is specifically indexed as GDP PPP per capita (current international $ for purchasing power parity) in 2014. GDP PPP is included as the index of economic affluence in this study as it has the advantage over GDP per capita because it considers the relative cost of local goods, services and inflation rates of the country. Therefore, GDP PPP is more associated with the life quality and wellbeing of individuals who live in different countries [7, 23, 24].

4. Obesity prevalence rate, measured with the percentage of adult individuals with the body mass index (BMI) equal to or exceeding 30 kg/m^2 in 2014 [18]

Obesity is a result of metabolic imbalances which increases the risk for obese individuals to develop medical complications and die younger than 65 years old [25].

5. Physician density, expressed with the number of physicians per 1,000 people [26]

Physician healthcare access determines not only the level of primary healthcare services, but also the diagnosis and treatment of patients' health conditions at the secondary and tertiary levels. Therefore, physician and nursing healthcare services may be seen to confound with each other for maintaining and improving people's health to facilitate them to survive 65 years old [27].

6. Fertility rate (total), representing the total number of children born to a woman [28]

It is reasonable to note that falling fertility rates are one of the major determinants of population aging [3-6]. Low fertility rates result in smaller youth cohorts, which creates an imbalance in the demographic structure: older age groups expand and become more populous than their younger counterparts [29].

7. Urbanization, indexed with the percentage of total population living in urban areas in 2014 [30]

Urbanization represents a major demographic shift which is characterised by lifestyle changes, for instance lack of physical activity and poor diet patterns [31-33]. Studies controversially showed that people living in both metropolitan areas and rural areas have greater life expectancy and higher percentage to survive 65 years old. However, urban residents experience relatively larger gains in life expectancy than those in rural areas [34].

The relevant United Nations agencies offer free online access to data required for the analyses such as in this study. The data can only be identifiable to the level of population. They are not identifiable or re-identifiable to the individual participant, their family or community. Therefore, there is no need to obtain ethical approval or consent during the entire study process.

Data Selection

We extracted the country specific data on independent variable (nurse density), dependent variable (population ageing level) and 5 potential confounders (GDP PPP, obesity prevalence, physician density, total fertility rate and urbanization). For each variable, we extracted the data from all the countries where data were available from the websites of the United Nation agencies.

Following the country list created by the World Bank, we aligned all the 7 variables from the individual countries and obtained a full set of data comprising 215 countries. Each country was treated as an individual study subject in all the data analysis models. However, not all the countries (subjects) have all the information for all the variables. The numbers of countries/ subjects (sample size) included for analysing the correlations to other variables may differ as such.

Data analysis

To assess the correlation between the nurse density and the 65yo%, the analysis proceeded in the following 5 models [27] [27, 35-38]:

1) Scatter plots were explored with the raw data in Microsoft Excel® for examining and visualizing the strength, shape and direction of correlation of nurse density to 65yo%. Additionally, the scatter plots graph allowed an examination of data quality, for example, if there are any unexpected gaps in the data and if there are any extreme outlier points.

To reduce the skewness of our original data for valid statistical analysis results produced in the following data analysis models, all the 7 variables were log-transformed.

2. Bivariate (Pearson's r and nonparametric) correlations were conducted to examine the directions and strengths of the correlations between all the variables.

3. Partial correlation of Pearson's moment-product approach was performed to identify the independent correlation relationships between variables. We alternated each of the 6 variables (nurse density, GDP PPP, obesity, physician density, total fertility rate and urbanization) as the independent predictor when all the other 5 variables are included as the potential confounding factors. And then, we alternated each individual variable as the controlled variable to assess the relationship between 65yo% and each of the 5 variables.

4. Standard multiple linear regression (enter) was performed to describe the correlations between the dependent variable (65yo%) and the predicting variables. To explore if and how much nurse density could statistically explain the individual relationships between nurse density and GDP PPP, obesity, physician density, total fertility rate and urbanization, the enter multiple linear regression was performed to calculate the correlations between nurse density and the confounding variables when nurse density is "added" and "not added" as a predicting variable respectively.

Subsequently, standard multiple linear regression (stepwise) is performed to select the most significant predicting variable(s) for 65yo% when nurse density was "added" and "not added" as a predicting variable respectively.

5. The universal correlations between nurse density and 65yo% were explored and compared in different country groupings:

1) the World Bank income classifications: high income, upper middle income, low-middle income and low income;

2) the UN common practice on defining the developed and developing countries [39];

Fisher's r-to-z transformation was applied to compare the nurse density- 65yo% correlations in the developed countries and the developing countries.

3) the WHO regional classifications: Africa (AFR), Americas (AMR), Eastern Mediterranean (EMR), Europe (EU), South-East Asia (SEAR) and Western Pacific (WPR) [40];

4) countries with the strong contrast in terms of geographic distributions, per capita GDP levels and/or cultural backgrounds. We analysed the correlation in the 6 country groupings: Asia Cooperation Dialogue (ACD) [41]; the Asia-Pacific Economic Cooperation (APEC); the Arab World [42], European Economic Area (EEA) [43], countries with English as the official language (government websites),

European Union (EU) [44], Latin America [45], Latin America and the Caribbean (LAC) [45], the Organisation for Economic Co-operation and Development (OECD) and non-OECD group [46].

Bivariate correlations, partial correlation, multiple linear regression analyses (enter and stepwise) were conducted with SPSS v. 28. The significance is kept at the 0.05 level, but 0.01 and 0.001 levels ae also reported. Standard multiple linear regression analysis criteria are set at probability of F to enter ≤ 0.05 and probability of F to remove ≥ 0.10.

Results

The relationship identified in the scatterplots between nurse density and population ageing was a strong correlation (R^2= 0.5268 (r= 0.7258), p<0.001, n= 177, Figure 1). The nursing workforce explains 52.68% of population ageing variance.

Figure 1 The relationship between nurse density and population ageing level

This strong relationship between nurse density and population ageing identified in the scatterplots was confirmed by the subsequent bivariate correlation analyses with the log-transformed data.

Globally, nurse density significantly correlated to 65yo% (r= 0.694 and rho= 0.724, p<0.001 respectively in Pearson and non-parametric analyses, Table 1).

Table 1 Pearson (above diagonal) & non-parametric (below diagonal) correlation matrix for all variables

	Nurse density	Population ageing	GDP PPP	Obesity prevalence	Physician density	Total fertility rate	Urbanization
Nurse density	1	0.694***	0.774***	0.508***	0.784***	-0.735***	0.547***
Population ageing	0.724***	1	0.671***	0.365***	0.759***	-0.852***	0.494***
GDP PPP	0.797***	0.686***	1	0.502***	0.839***	-0.799***	0.720***
Obesity prevalence	0.465***	0.365***	0.483***	1	0.500***	-0.391***	0.546***
Physician density	0.810***	0.810***	0.831***	0.445***	1	-0.841***	0.626***
Total fertility rate	-0.745***	-0.872***	-0.777***	-0.352***	-0.816***	1	-0.521***
Urbanization	0.600***	0.506***	0.757***	0.584***	0.630***	-0.522***	1

Significance level: ***p< 0.001; n ranges between 176 and 212.

Data source and definition: Nurse density, expressed with the number of nurses and midwives per 1,000 population (the Word Bank); Population ageing, presented with the percent of population aged 65 and above (the Word Bank); GDP PPP, the per capita purchasing power parity (PPP) value of all final goods and services produced within a territory in a given year (the Word Bank); Obesity prevalence, the percentage of population with BMI ≥30 prevalence (WHO Global Health Observatory); Physician availability, the number of nurses and midwives per 1,000 population (the Word Bank); Total fertility rate, representing the number of children that are born to a woman, the World Bank; Urbanization, the percentage of population living in urban area (the Word Bank);

All the data were log-transformed for correlation analysis.

When predicting and confounding variables (nurse density, GDP PPP, obesity, total fertility rate, physician density and urbanization) were individually correlated to population ageing, while keeping the other 5 variables statistically constant, nurse density, GDP PPP and total fertility rate remained significant correlations to 65yo% (r= 0.194, p< 0.05; r= 0.223, p< 0.01 and r= 0.609, p < 0.001 respectively, Table 2-1). Neither obesity prevalence nor urbanization showed an independent correlation to 65yo% while the other 5 variables were kept statistically constant (Table 2-1).

Moreover, nurse density was in constant and significant correlation to 65yo% when GDP PPP, obesity prevalence, total fertility rate, physician density and urbanization were individually controlled ($r = 0.371$, $p < 0.001$; $r = 0.634$, $p < 0.001$; $r = 0.243$, $p < 0.001$; $r = 0.189$, $p < 0.01$ and $r = 0.582$, $p < 0.001$ respectively, Table 2-2).

These partial correlation analysis results suggest that, statistically, the individual, or the combined confounding effects of GDP PPP, obesity prevalence, total fertility rate, physician density and urbanization did not affect the significant relationship of nurse density to people increasing over 65yo%.

Table 2 Partial correlation coefficients between Population ageing and nurse density with individual and different combinations of controlled variables

Table 2-1: Nurse density, GDP PPP, obesity prevalence, physician density, total fertility rate and urbanization were alternated as the predicting variable for calculating its relationship with population ageing level while the other 4 variables were kept statistically constant.

Variables	Population ageing (65yo%)		Population ageing (65yo%)		Population ageing (65yo%)		Population ageing (65yo%)		Population ageing (65yo%)		Population ageing (65yo%)	
	r	p	r	p	r	p	r	p	r	p	r	p
Nurse density	0.194	<0.050	-	-	-	-	-	-	-	-	-	-
GDP PPP	-	-	-0.223	<0.010	-	-	-	-	-	-	-	-
Obesity prevalence	-	-	-	-	-0.038	0.626	-	-	-	-	-	-
Physician density	-	-	-	-	-	-	0.119	0.120	-	-	-	-
Total fertility rate	-	-	-	-	-	-	-	-	-0.609	< 0.001	-	-
Urbanization	-	-	-	-	-	-	-	-	-	-	0.147	0.055

Table 2-2: Nurse density, GDP PPP, obesity prevalence, physician density, total fertility rate and urbanization were alternated as the individual potential confounder for exploring the partial correlations between population ageing level and the other 4 independent/confounding variables.

Variables	Population ageing (65yo%)		Population ageing (65yo%)		Population ageing (65yo%)		Population ageing (65yo%)		Population ageing (65yo%)		Population ageing (65yo%)	
	r	p	r	p	r	p	r	p	r	p	r	p
Nurse density	-	-	0.371	< 0.001	0.634	< 0.001	0.243	< 0.001	0.189	< 0.010	0.582	< 0.001
GDP PPP	0.295	< 0.001	-	-	0.606	< 0.001	0.097	0.196	-0.031	0.683	0.523	< 0.001
Obesity prevalence	0.020	0.793	0.043	0.569	-	-	-0.027	0.720	0.064	0.394	0.130	0.084
Physician density	0.482	< 0.001	0.487	< 0.001	0.716	< 0.001	-	-	0.150	0.043	0.664	< 0.001
Total fertility rate	-0.701	< 0.001	-0.709	< 0.001	-0.828	< 0.001	-0.607	< 0.001	-	-	-0.802	< 0.001
Urbanization	0.190	< 0.010	0.021	0.782	0.378	< 0.001	0.036	0.629	0.111	0.129	-	-

- Controlled variable; Table 2-1 df = 169; Table 2-2 df range: 173 to 188

Data source and definition Data source and definition: Nurse density, expressed with the number of nurses and midwives per 1,000 population (the Word Bank); Population ageing, presented with the percent of population aged 65 and above (the Word Bank); GDP PPP, the per capita purchasing power parity (PPP) value of all final goods and services produced within a territory in a given year (the Word Bank); Obesity prevalence, the percentage of population with BMI ≥30 prevalence (WHO Global Health Observatory); Physician availability, the number of nurses and midwives per 1,000 population (the Word Bank); Total fertility rate, representing the number of children that are born to a woman, the World Bank; Urbanization, the percentage of population living in urban area (the Word Bank);

All the data were log-transformed for correlation analysis.

Standard multiple linear regression (enter) analysis was applied to further predict 65yo% when nurse density, GDP PPP, obesity, physician density and urbanization were considered as the predicting variables. When nurse density was "not added" as one of the predicting variables, physician density and total fertility rate were in significant correlations to 65yo% (β= 0.230, p<0.01 and β= -0.770, p<0.001 respectively). GDP PPP, obesity prevalence and urbanization showed weak and insignificant correlations to 65yo% (Table 3-1). When nurse density was "added" as a predicting variable, it showed its significant contribution to 65yo% together with total fertility rate (β= 0.178, p<0.010; β= -0.239, p<0.010; β= -0.749, p< 0.001, Table 3-1). Obesity prevalence, physician density and urbanization showed very weak and insignificant relationship to 65yo% (Table 3-1).

Similarly, in the subsequent stepwise linear regression model, when nurse density was "not added" as one of the predictors, total fertility rate and physician density were selected as the two most significant variables which totally explained 72.9% (R^2 = 0.729, Table 3-2) of 65yo% variance. While nurse density was "added" as a predicting variable, the stepwise linear regression model selected nurse density as the 2nd most influential predictor for 65yo% with (R^2 increment= 0.010, Table 3-2). In the stepwise regression analysis model, totally, 74.1% (R^2= 0.741) of 65yo% variance was explained by the 4 selected significant predicting and potential confounding variables (total fertility rate, nurse density, GDPP PPP and physician density, Table 3-2).

Table 3 Multiple linear regression results to show predicting effects of independent variables and identify the significant predictors of population ageing level

Enter

	Population ageing (65yo%)			
	Nurse density (not added)		Nurse density (added)	
Variable	Beta	Significance	Beta	Significance
Nurse density	Not added		0.178	< 0.050
GDP PPP	-0.105	0.065	-0.239	< 0.010
Obesity prevalence	0.029	0.563	0.008	0.874
Physician density	0.230	< 0.010	0.166	0.069
Total fertility rate	-0.770	< 0.001	-0.749	< 0.001
Urbanization	0.011	0.857	0.032	0.607

Stepwise

Population ageing (65yo%)					
Nurse density (not added)			Nurse density (added)		
Model	Variable	Adjusted R^2	Model	Variable	Adjusted R^2
1	Total fertility rate	0.722	1	Total fertility rate	0.722
2	Physician density	0.729	2	Nurse density	0.732
	GDP PPP	Insignificant	3	GDP PPP	0.736
	Obesity prevalence	Insignificant	4	Physician density	0.741
	Urbanization	Insignificant		Obesity prevalence	Insignificant
	Nurse density	Not added		Urbanization	Insignificant

Significance level: * p<0.05; ** p< 0.01; ***p< 0.001

Data source and definition Data source and definition: Nurse density, expressed with the number of nurses and midwives per 1,000 population (the Word Bank); Population ageing, presented with the percent of population aged 65 and above (the Word Bank); GDP PPP, the per capita purchasing power parity (PPP) value of all final goods and services produced within a territory in a given year (the Word Bank); Obesity prevalence, the percentage of population with BMI ≥30 prevalence (WHO Global Health Observatory); Physician availability, the number of nurses and midwives per 1,000 population (the Word Bank); Total fertility rate, representing the number of children that are born to a woman, the World Bank; Urbanization, the percentage of population living in urban area (the Word Bank);

All the data were log-transformed for correlation analysis.

Table 4 showed the relationship between nurse density and 65yo% in different country groupings. The best fit equations and R square extracted from the scatt plots consisting of nurse density and 65yo% showed that nurse density universally correlated to 65yo% in all country groupings. The highlights of the results were the relationships between nurse density and 65yo% in United Nations developed and developing countries (R^2= 0.722 and 0.248 respectively, Table 4). Fisher's r to z transformation revealed that nurse density had the significantly more important role in promoting 65yo% in developed countries than in the developing countries (z= 4.02, p< 0.001, Table 4).

Table 4 Bivariate correlations between Nurse density and population ageing level within various country groupings

**Country grouping**	**Best fit equation**	R^2	**Trendline pattern**	**Fisher's r-to-z transformation**
UN common practice				
Developed	$y = -0.4141x^2 + 1.7808x + 1.0889$	$R^2 = 0.722$, n=45	Polynomial	Developed vs developing countries: z= 4.02, p< 0.001
Developing	$y = 0.0167x^2 + 0.0891x + 0.6085$	$R^2 = 0.248$, n=141	Polynomial	
World Bank income classifications				
Low	$y = 0.0814x^2 - 0.6136x + 2.1834$	$R^2 = 0.2908$	Polynomial	
Low middle	$y = -0.0249x^2 + 0.5036x - 0.4412$	$R^2 = 0.1924$	Polynomial	
Upper middle	$y = 0.0381x^2 - 0.1774x + 1.7187$	$R^2 = 0.1819$	Polynomial	
High	$y = -0.1445x^2 + 2.5845x - 8.3844$	$R^2 = 0.1590$	Polynomial	
WHO Regions				
AFRO	$y = 0.1026x^2 - 0.815x + 2.6237$	$R^2 = 0.4013$	Polynomial	
AMRO	$y = 0.0176x^2 + 0.0354x + 1.3525$	$R^2 = 0.4841$	Polynomial	
EMRO	$y = -0.167x^2 + 1.6365x - 2.4014$	$R^2 = 0.3026$	Polynomial	
EURO	$y = -0.1331x^2 + 0.8006x + 1.6858$	$R^2 = 0.0552$	Polynomial	
SEARO	$y = -0.1309x^2 + 1.4553x - 2.083$	$R^2 = 0.1015$	Polynomial	
WPRO	$y = 0.18x^2 - 1.634x + 5.0363$	$R^2 = 0.6539$	Polynomial	
Countries grouped based on various factors				
ACD	$y = 0.6558\ln(x) + 0.583$	$R^2 = 0.0359$	Logarithmic	
APEC	$y = 0.6242e^{0.2021x}$	$R^2 = 0.6200$	Exponential	
Arab World	$y = -0.143x^2 + 1.408x - 1.93$	$R^2 = 0.2206$	Polynomial	
EEA	$y = -0.2023x^2 + 0.957x + 1.8014$	$R^2 = 0.3603$	Polynomial	
EOL	$y = 0.1063x^2 - 0.7988x + 2.4714$	$R^2 = 0.5424$	Polynomial	
EU	$y = -0.1635x^2 + 0.6147x + 2.4294$	$R^2 = 0.0776$	Polynomial	
LA	$y = 0.0263x^2 - 0.0409x + 1.4964$	$R^2 = 0.6468$	Polynomial	
LAC	$y = 0.0031x^2 + 0.1594x + 1.1142$	$R^2 = 0.4057$	Polynomial	
OECD	$y = -0.2023x^2 + 0.957x + 1.8014$	$R^2 = 0.3603$	Polynomial	
SADC	$y = 0.1063x^2 - 0.7988x + 2.4714$	$R^2 = 0.5424$	Polynomial	

Data source and definition: Nurse density, measured with the number of nurses and midwives per 1,000 population (the Word Bank); Population ageing, presented with the percent of population aged 65 and above (the Word Bank).

All the data were log-transformed for correlation analysis.

Discussion

This ecological study examined the correlation between nursing density and population ageing (65yo%) with consideration of the confounding effects of economic affluence (GDP PPP), obesity prevalence,

physician density, total fertility rate and urbanization. The findings in this study reveal that nurse density may be a significant contributor to population ageing, and this contributing effect remains independent of economic affluence (GDP PPP), total fertility rate, obesity, physician density and urbanization.

It is well-known that, as the biggest healthcare professional group [10], nurses are essential to primary, secondary and tertiary health care. This is evident in the intrinsic correlation between nurse density and improvements to life expectancies at birth and 65 years of age, old within OECD countries [13] and worldwide [12].

The impact of nursing has been seen in many domains of health such as management of infectious diseases, a significant contributor to reduced life expectancy. As healthcare professionals in constant communication with patients and their needs nurses have led and manage infection control services across acute and community settings, effectively interrupting disease transmission on multiple levels of population and social status [47, 48]. As the frontline healthcare professional, nurses have been playing a crucial role in preventing COVID- 19 from spreading and treating COVID-19 patients [49, 50]. Nursing interventions have been reducing infectious disease mortality rates across the whole population, including the segment aged 65 and over [51].

High child mortality rates and the ongoing impact of an infectious disease pandemic are significant obstacles for people to survive to 65 years of age. Globally, nurses have been playing a pivotal role in reducing child mortality rate through heavy involvement in communicable and non-communicable disease management [11, 52, 53]. Thompson and Keeling report that over 40 years, improvements in child mortality have occurred through nurses educating and assisting young mothers on how to feed their children and how to maintain hygiene [52].

Patient education and public health promotion are critical components of the nurses' daily job, which may be even more important concerns for the older population because of the second epidemiology transition [54]. This transition, marked by a rapidly growing public health crisis concerning a shift from infectious diseases to the impact of chronic diseases is a consequence of good nursing care. This has been evidenced in the role of the nursing workforce in contributing to life expectancy both at younger age and then older than 65 across epidemics, acute health conditions [12, 13] and a range of degenerative diseases which occur in older populations [13]. In terms of approach, both studies also correlated nurse density to life expectancy at population level, similar to those revealed in this study.

Nurses are central to full coverage healthcare services, they are often the first and sometimes the only health professionals that patients see [10]. The quality of healthcare received from nurses has been associated with patient safety [55-60], low mortality rate [61-66] and patient outcomes [55, 59, 67-70]. While the breadth of the impact of nurses on people's health remains difficult to fully quantify, the community recognition of nursing healthcare services occurs through various awards, for example, the International Year of the Nurse and the Midwife [71] and the Gallup Roll [72].

Strengths and limitations

The greatest strength of this study is free and unrestricted access to the predicting variable (nurse density) and dependent variable (65yo%) which was complimented with data concerning potential confounding variables (GDP PPP, obesity prevalence, total fertility rate and urbanization). The time based serial data enabled both predicting and confounding variables to observe their delayed presentations and impact on 65yo%. This enabled data analysis using several different models, such as scatterplots, bivariate, partial correlation and linear regression. This level of data availability and subsequent multiple data analysis approaches provide a rich source of findings when compared to individual based studies.

Some limitations to be noted:

Firstly, this is an ecological study, and the results are subject to ecological fallacy. The correlations identified in this study are at population level, but they may not necessarily hold true at the individual level.

Secondly, this is a cross sectional study, the relationship between nurse density and 65yo% is only correlational, not causal.

Thirdly, the data included in this study may be crude and have some random errors when the United Nations agencies collected and aggregated data at population level. However, the analysis results are highly repeatable which may be different from individual data based studies.

Implications for Practice

This study delivers a strong message to health authorities worldwide. As populations age 65 and over increase, the prevalence of disability, frailty and chronic diseases also rises [14, 73, 74]. With the increasing percentage of the world population aged 65+, the pressure for nursing care becomes cumulative, essentially exacerbating the challenges of a shortage of nursing staff. This study should encourage healthcare authorities, to consider the implications increase of density when promoting population health.

Conclusions

Nurse density correlates to population ageing globally and regionally, but is more strongly in developed countries. This suggests that nurse density may be a significant predictor for population ageing, and is more prominent in developed countries. Ironically, nursing healthcare has been partially responsible for population ageing which in turn demands more nursing care and accordingly exposes the nursing profession to a worsening shortage of staff. To highlight the profound implications for the role the nursing profession plays especially at a time of global nursing shortage, further study into the effects of long-run elasticity of nurse staffing level on population ageing may be needed. For instance, what percentage of increase of nursing staff would be required to meet every 1% increase of an ageing population.

Authors' contributions

WY and FD contributed to the study design. Both authors conducted the data analysis and interpreted data analysis results. WY drafted the manuscript with input from FD. Both authors contributed, read and approved the final manuscript.

Conflicts of Interest

The author declares no conflict of interest.

References

1. United Nations. Ageing Official website: United Nations; 2022. Available from: https://www.un.org/en/global-issues/ageing.

2. OCED Data. Demography- Elderly population: Organisation for Economic Co-operation and Development; 2022 [November 12 2022]. Available from: https://data.oecd.org/pop/elderly-population.htm.

3. Lee R, Mason A, network N, Lee R, Mason A, Amporfu E, et al. Is low fertility really a problem? Population aging, dependency, and consumption. Science. 2014;346(6206):229-34. doi: https://doi.org/10.1126/science.1250542.

4. ESHRE Capri Workshop Group. Fertility and ageing. Human reproduction update. 2005;11(3):261-76. doi: https://doi.org/10.1093/humupd/dmi006.

5. Cheng X, Yang Y, Schwebel DC, Liu Z, Li L, Cheng P, et al. Population ageing and mortality during 1990–2017: a global decomposition analysis. PLoS medicine. 2020;17(6):e1003138. doi: https://doi.org/10.1371/journal.pmed.1003138.

6. Harper S. Living longer within ageing societies. Journal of Population Ageing. 2019;12(2):133-6. doi: https://doi.org/10.1007/s12062-019-09248-4.

7. Marmot MG, Smith GD. Why are the Japanese living longer? BMJ: British Medical Journal. 1989;299(6715):1547. doi: https://doi.org/10.1136/bmj.299.6715.1547.

8. Hummer RA, Hernandez EM. The effect of educational attainment on adult mortality in the United States. Population bulletin. 2013;68(1):1-16. doi: https://www.ncbi.nlm.nih.gov/pmc/articles/PMC4435622/. PubMed Central PMCID: PMC4435622.

9. Timmers PR, Wilson JF, Joshi PK, Deelen J. Multivariate genomic scan implicates novel loci and haem metabolism in human ageing. Nature communications. 2020;11(1):1-10. doi: https://doi.org/10.1038/s41467-020-17312-3.

10. WHO. Nursing and midwifery Internet The World Health Organization; 2020 [January 8 2022]. Available from: https://www.who.int/news-room/fact-sheets/detail/nursing-and-midwifery.

11. Amiri A, Vehviläinen-Julkunen K, Solankallio-Vahteri T, Tuomi S. Impact of nurse staffing on reducing infant, neonatal and perinatal mortality rates: Evidence from panel data analysis in 35 OECD countries. International journal of nursing sciences. 2020;7(2):161-9. doi: https://doi.org/10.1016/j.ijnss.2020.02.002.

12. You W, Donnelly F. Although in shortage, nursing workforce is still a significant contributor to life expectancy at birth. Public Health Nursing. 2022. doi: https://doi.org/10.1111/phn.13158.

13. Amiri A, Solankallio-Vahteri T. Nurse staffing and life expectancy at birth and at 65 years old: evidence from 35 OECD countries. International journal of nursing sciences. 2019;6(4):362-70. doi: https://doi.org/10.1016/j.ijnss.2019.07.001.

14. WHO. Ageing and health: The World Health Orgnization 2022 [18 December 2022]. Available from: https://www.who.int/news-room/fact-sheets/detail/ageing-and-health.

15. Tamata AT, Mohammadnezhad M. A systematic review study on the factors affecting shortage of nursing workforce in the hospitals. Nursing open. 2023;10(3):1247-57. doi: https://doi.org/10.1002/nop2.1434.

16. Xu H, Intrator O, Bowblis JR. Shortages of staff in nursing homes during the COVID-19 pandemic: what are the driving factors? Journal of the American Medical Directors Association. 2020;21(10):1371-7. doi: https://doi.org/10.1016/j.jamda.2020.08.002.
17. The World Bank. How does the World Bank classify countries? : The World Bank; 2022 [December 24 2022]. Available from: https://datahelpdesk.worldbank.org/knowledgebase/articles/378834-how-does-the-world-bank-classify-countries.
18. WHO. GHO | By category | Obesity (body mass index ≥ 30),age-standardized (%) - Estimates by country: World Health Organization; 2017 [updated MINERVA PUBLISH DATE28 August 2017]. Available from: http://apps.who.int/gho/data/view.main.CTRY2450A.
19. The World Bank. DataBank Official website2022 [May 18 2022]. Available from: https://databank.worldbank.org/home.aspx.
20. The world Bank. Population ages 65 and above (% of total population) Officiel website: The World Bank; 2020 [cited 2022 16 May 2022]. Available from: https://data.worldbank.org/indicator/SP.POP.65UP.TO.ZS.
21. World Bank. Nurses and midwives (per 1,000 people) DataBank: the World Bank Group; 2022 [January 18 2022]. Available from: https://data.worldbank.org/indicator/SH.MED.NUMW.P3.
22. World Bank. GDP per capita, PPP (current international $) Internet2019 [December 28 2021]. Available from: https://data.worldbank.org/indicator/NY.GDP.PCAP.PP.CD.
23. Becker GS, Philipson TJ, Soares RR. The quantity and quality of life and the evolution of world inequality. American economic review. 2005;95(1):277-91. doi: https://doi.org/10.1257/0002828053828563.
24. Deaton A. Income, health, and well-being around the world: Evidence from the Gallup World Poll. Journal of Economic perspectives. 2008;22(2):53-72. doi: https://doi.org/10.1257/jep.22.2.53.
25. Peeters A, Barendregt JJ, Willekens F, Mackenbach JP, Al Mamun A, Bonneux L. Obesity in adulthood and its consequences for life expectancy: a life-table analysis. Annals of internal medicine. 2003;138(1):24-32. doi: https://doi.org/10.7326/0003-4819-138-1-200301070-00008.
26. World Bank. Physicians (per 1,000 people) Online: The World Bank; 2020 [April 3 2022]. Available from: https://data.worldbank.org/indicator/SH.MED.PHYS.ZS.
27. You W, Donnelly F. Physician care access plays a significant role in extending global and regional life expectancy. European Journal of Internal Medicine. 2022;(103):62-8. doi: https://doi.org/10.1016/j.ejim.2022.06.006.
28. The world Bank. Fertility rate, total (births per woman) Official website: The World Bank; 2015 [16 May 2022]. Available from: https://data.worldbank.org/indicator/SP.DYN.TFRT.IN.
29. Guseh JS. Aging of the World's Population. Encyclopedia of Family Studies. 2016:1-5. doi: https://doi.org/10.1002/9781119085621.wbefs352.
30. World Bank. Urban population (% of total population) Official Website: The World Bank Group; 2022 [January 18 2022]. Available from: https://data.worldbank.org/indicator/SP.URB.TOTL.IN.ZS.
31. Allender S, Foster C, Hutchinson L, Arambepola C. Quantification of urbanization in relation to chronic diseases in developing countries: a systematic review. Journal of urban health : bulletin of the New York Academy of Medicine. 2008;85(6):938-51. Epub 2008/10/22. doi: https://doi.org/10.1007/s11524-008-9325-4.

32. Moore M, Gould P, Keary BS. Global urbanization and impact on health. International journal of hygiene and environmental health. 2003;206(4):269-78. doi: https://doi.org/10.1078/1438-4639-00223.
33. WHO. Urban health WHO Official Website: World Health Organization; 2022 [March 12 2022]. Available from: https://www.who.int/health-topics/urban-health#tab=tab_1.
34. Singh GK, Siahpush M. Widening rural–urban disparities in life expectancy, US, 1969–2009. American journal of preventive medicine. 2014;46(2):e19-e29. doi: https://doi.org/10.1016/j.amepre.2013.10.017.
35. You W, Henneberg R, Coventry BJ, Henneberg M. Cutaneous malignant melanoma incidence is strongly associated with European depigmented skin type regardless of ambient ultraviolet radiation levels: evidence from Worldwide population-based data. AIMS Public Health. 2022;9(2):378. doi: https://doi.org/10.3934/publichealth.2022026.
36. You W, Henneberg R, Henneberg M. Healthcare services relaxing natural selection may contribute to increase of dementia incidence. Scientific Reports. 2022;12(1):1-10. doi: https://doi.org/10.1038/s41598-022-12678-4.
37. You W, Rühli F, Eppenberger P, Galassi FM, Diao P, Henneberg M. Gluten consumption may contribute to worldwide obesity prevalence. Anthropological Review. 2020;83(3):327-48. doi: http://dx.doi.org/10.2478/anre-2020-0023.
38. You W, Henneberg M. Large household reduces dementia mortality: A cross-sectional data analysis of 183 populations. PloS one. 2022;17(3):e0263309. doi: https://doi.org/10.1371/journal.pone.0263309.
39. United Nations Statistics Division. Composition of macro geographical (continental) regions, geographical sub-regions, and selected economic and other groupings: United Nations; 2013 [03.10.2022]. Available from: http://unstats.un.org
40. WHO. WHO regional offices: The World Health Orgnization 2018 [November 26.2022]. Available from: http://www.who.int.
41. Asia Cooperation Dialogue. Member Countries 2018. Available from: http://www.acddialogue.com.
42. The World Bank. Arab World | Data: The World Bank; 2015. Available from: http://data.worldbank.org/region/ARB.
43. European Free Trade Association. What is the European Economic Area? 2022. Available from: http://www.efta.int/eea.
44. European Union. EUROPA - Countries 2022 [18.06.2015]. Available from: http://europa.eu.
45. The United Nations Educational Scientific and Cultural Organization. UNESCO Regions-Latin America and the Caribbean 2022 [[11.26.2015]. Available from: http://www.unesco.org.
46. OECD. List of OECD Member countries 2015. Available from: http://www.oecd.org.
47. You W, Galassi FM, Varotto E, Henneberg M. Genghis Khan's death (AD 1227): An unsolvable riddle or simply a pandemic disease? International Journal of Infectious Diseases. 2021;104:347-8. doi: https://doi.org/10.1016/j.ijid.2020.12.089.
48. Lam SKK, Kwong EWY, Hung MSY, Chien WT. Emergency nurses' perceptions regarding the risks appraisal of the threat of the emerging infectious disease situation in emergency departments. International journal of qualitative studies on health and well-being. 2020;15(1):1718468. doi: https://doi.org/10.1080/17482631.2020.1718468.
49. Al Thobaity A, Alshammari F. Nurses on the frontline against the COVID-19 pandemic: an integrative review. Dubai Medical Journal. 2020;3(3):87-92. doi: https://doi.org/10.1159/000509361.

50. Fawaz M, Anshasi H, Samaha A. Nurses at the front line of COVID-19: Roles, responsibilities, risks, and rights. The American journal of tropical medicine and hygiene. 2020;103(4):1341. doi: https://doi.org/10.4269/ajtmh.20-0650.
51. Stirling B, Littlejohn P, Willbond ML. Nurses and the Control of Infectious Disease. Understanding epidemiology and disease transmission is vital to nursing care. Canadian Nurse. 2004;100(9):16-20. PubMed Central PMCID: PMC15623008.
52. Thompson ME, Keeling AA. Nurses' Role in the Prevention of Infant Mortality in 1884–1925: Health Disparities Then and Now. Journal of pediatric nursing. 2012;27(5):471-8. doi: https://doi.org/10.1016/j.pedn.2011.05.011.
53. Amiri A. Role of nurses and midwives in improving patient safety during childbirth: evidence from obstetric trauma in OECD countries. Applied Nursing Research. 2020;56:151343. doi: https://doi.org/10.1016/j.apnr.2020.151343.
54. McKeown RE. The Epidemiologic Transition: Changing Patterns of Mortality and Population Dynamics. Am J Lifestyle Med. 2009;3(1 Suppl):19S-26S. Epub 2010/02/18. doi: https://doi.org/10.1177/1559827609335350. PubMed PMID: 20161566; PubMed Central PMCID: PMC2805833.
55. Aiken LH, Sermeus W, Van den Heede K, Sloane DM, Busse R, McKee M, et al. Patient safety, satisfaction, and quality of hospital care: cross sectional surveys of nurses and patients in 12 countries in Europe and the United States. BMJ. 2012;344(e1717). doi: https://doi.org/10.1136/bmj.e1717.
56. Hughes RG. Chapter 44 Tools and strategies for quality improvement and patient safety. Patient safety and quality: An evidence-based handbook for nurses Rockville (MD): Agency for Healthcare Research and Quality (US). 2008. doi: https://www.ncbi.nlm.nih.gov/books/NBK2682/.
57. Wu Y, Fujita S, Seto K, Ito S, Matsumoto K, Huang C-C, et al. The impact of nurse working hours on patient safety culture: a cross-national survey including Japan, the United States and Chinese Taiwan using the Hospital Survey on Patient Safety Culture. BMC health services research. 2013;13(1):1-7. doi: https://doi.org/10.1186/1472-6963-13-394.
58. Ausserhofer D, Zander B, Busse R, Schubert M, De Geest S, Rafferty AM, et al. Prevalence, patterns and predictors of nursing care left undone in European hospitals: results from the multicountry cross-sectional RN4CAST study. BMJ quality & safety. 2014;23(2):126-35. doi: https://doi.org/10.1136/bmjqs-2013-002318.
59. Cho E, Lee N-J, Kim E-Y, Kim S, Lee K, Park K-O, et al. Nurse staffing level and overtime associated with patient safety, quality of care, and care left undone in hospitals: a cross-sectional study. International journal of nursing studies. 2016;60:263-71. doi: https://doi.org/10.1016/j.ijnurstu.2016.05.009.
60. Amiri A, Solankallio-Vahteri T, Tuomi S. Role of nurses in improving patient safety: evidence from surgical complications in 21 countries. International journal of nursing sciences. 2019;6(3):239-46. doi: https://doi.org/10.1016/j.ijnss.2019.05.003.
61. Aiken LH, Clarke SP, Sloane DM, Sochalski J, Silber JH. Hospital nurse staffing and patient mortality, nurse burnout, and job dissatisfaction. Jama. 2002;288(16):1987-93. doi: https://doi.org/10.1001/jama.288.16.1987.
62. Aiken LH, Cimiotti JP, Sloane DM, Smith HL, Flynn L, Neff DF. Effects of nurse staffing and nurse education on patient deaths in hospitals with different nurse work environments. The Journal of nursing administration. 2012;42(10 Suppl):S10. doi: https://doi.org/10.1097/MLR.0b013e3182330b6e.

63. Estabrooks CA, Midodzi WK, Cummings GG, Ricker KL, Giovannetti P. The impact of hospital nursing characteristics on 30-day mortality. Nursing research. 2005;54(2):74-84. doi: https://doi.org/10.1097/00006199-200503000-00002.
64. Van den Heede K, Lesaffre E, Diya L, Vleugels A, Clarke SP, Aiken LH, et al. The relationship between inpatient cardiac surgery mortality and nurse numbers and educational level: analysis of administrative data. International journal of nursing studies. 2009;46(6):796-803. doi: https://doi.org/10.1016/j.ijnurstu.2008.12.018.
65. Cho E, Sloane DM, Kim E-Y, Kim S, Choi M, Yoo IY, et al. Effects of nurse staffing, work environments, and education on patient mortality: an observational study. International journal of nursing studies. 2015;52(2):535-42. doi: https://doi.org/10.1016/j.ijnurstu.2014.08.006.
66. Manojlovich M. Increasing nurse staffing levels and a higher proportion with bachelor's degrees could decrease patient mortality risk. Evidence-based nursing. 2015;18(2):62-. doi: https://doi.org/10.1136/eb-2014-101913.
67. Oliver GM, Pennington L, Revelle S, Rantz M. Impact of nurse practitioners on health outcomes of Medicare and Medicaid patients. Nursing Outlook. 2014;62(6):440-7. doi: https://doi.org/10.1016/j.outlook.2014.07.004.
68. Aiken LH, Cerón C, Simonetti M, Lake ET, Galiano A, Garbarini A, et al. Hospital nurse staffing and patient outcomes. Revista Médica Clínica Las Condes. 2018;29(3):322-7. doi: https://doi.org/10.1016/j.rmclc.2018.04.011.
69. Rafferty AM, Clarke SP, Coles J, Ball J, James P, McKee M, et al. Outcomes of variation in hospital nurse staffing in English hospitals: cross-sectional analysis of survey data and discharge records. International journal of nursing studies. 2007;44(2):175-82. doi: https://doi.org/10.1016/j.ijnurstu.2006.08.003.
70. Suhonen R, Papastavrou E, Efstathiou G, Tsangari H, Jarosova D, Leino-Kilpi H, et al. Patient satisfaction as an outcome of individualised nursing care. Scandinavian journal of caring sciences. 2012;26(2):372-80. doi: https://doi.org/10.1111/j.1471-6712.2011.00943.x.
71. WHO. Year of the Nurse and the Midwife 2020 [28 June 2022]. Available from: https://www.who.int/campaigns/annual-theme/year-of-the-nurse-and-the-midwife-2020.
72. Oakland University. Gallup Poll finds nursing is most honest, ethical profession Internet: Oakland University; 2021. Available from: https://oakland.edu/oumagazine/news/nursing/2021/gallup-poll-finds-nursing-is-most-honest-ethical-profession.
73. Australian Institute of Health and Welfare. Australian Institute of Health and Welfare Feather Article 2014 [18 December 2022]. Available from: https://www.aihw.gov.au/getmedia/19dbc591-b1ef-4485-80ce-029ff66d6930/6_9-health-ageing.pdf.aspx.
74. National Academy of Sciences. Retooling for an Aging America: Building the Health Care Workforce. In: Americans. IoMUCotFHCWfO, editor. 2 Health Status and Health Care Service Utilization. Washington (DC): National Academies Press (US); 2008.

Figure
Click here to access/download;Figure;5 Fig 1 Nursing workforce n 65+.pdf

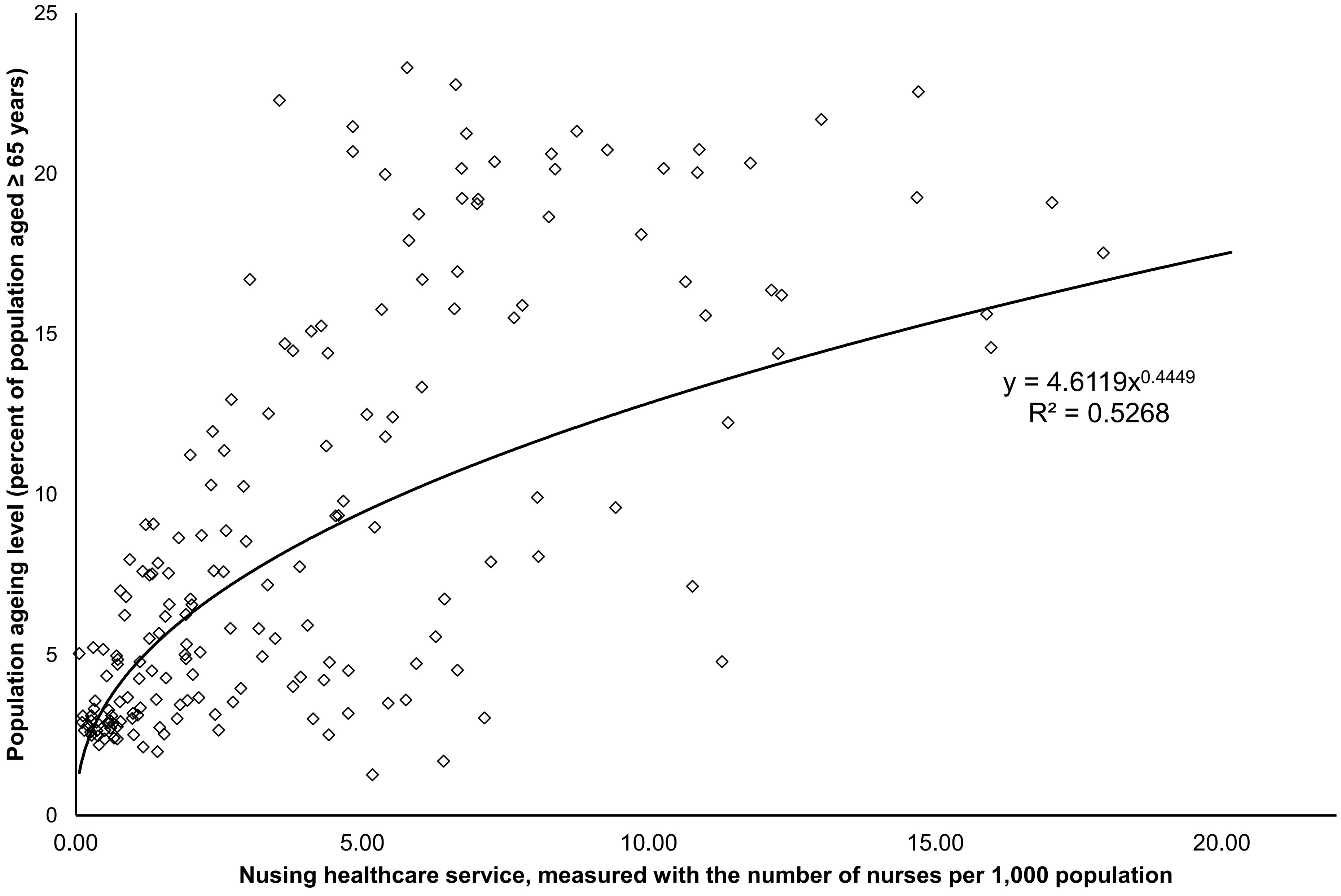

Greater nurse density correlates to higher level of population ageing globally, but is more prominent in developed countries.

Wenpeng You, PhD[1,2,3], Frank Donnelly, PhD[1]

[1] Adelaide Nursing School, the University of Adelaide, Adelaide, Australia

[2] Heart and Lung, Royal Adelaide Hospital, Adelaide, Australia

[3] Adelaide Medical School, the University of Adelaide, Adelaide, Australia

Running Head: Nurses promote population ageing

Corresponding author:
Wenpeng You, PhD, RN
wenpeng.you@adelaide.edu.au

Abstract

Background

Representing over 50% of the healthcare workforce, nurses provide care to people at all ages. This study advances, at a population level, that high levels of nursing services, measured by nurse density may significantly promote population ageing measured by the percentage of a population over 65 years of age (65yo%).

Methods

Population level data was examined to explore the correlation between nurse density and 65yo%. The confounding impacts on ageing such as the effects of economic affluence, physician density, fertility rate, obesity and urban advantages were also considered. Scatter plots, bivariate correlation, partial correlation and multiple linear regression analyses were performed for examining the correlations.

Results

Nurse density correlated to 65yo%; this relationship was independent of other influences such as fertility rate, economic affluence, obesity prevalence, physician density and urban advantages. Second to fertility rate, nursing density had the greatest influence on 65yo%. The predicting and confounding variables explain 74.4% of the total 65yo% variance. The universal correlations identified in country groupings suggest that low nurse density may be a significant global concern.

Conclusions

While nurse density might contribute significantly to 65yo% globally, the effect was more prominent in developed countries. Ironically, countries with higher nurse densities and therefore greater levels of 65yo%, were countries with an increased need for more nursing staff. ~~Findings such as this have~~To highlight the

profound implications for the role the nursing profession plays especially at a time of global nursing shortage. , further study into the effects of long-run elasticity of nurse staffing level on population ageing may be needed. For instance, what percentage of increase of nursing staff would be required to meet every 1% increase of an ageing population.

Keywords:

Nursing healthcare, Population ageing, Nursing staff shortage, Nursing workforce, Nurse density

Introduction

Over the last 200 years, people around the world have achieved impressive progress in health represented by an increase of the number of people aged 65 and over. Population ageing refers to changes in the age composition of a population which increases the proportion of older persons. It has become the 21st century's dominant demographic measure. Demographically, the most often used index of population ageing is the percentage of population segment aged 65 and over (65yo% hereafter) [1-3, 2].

It is well-established that declining fertility rates and rising life expectancy are the primary causes for population ageing [4-73-6]. Without sustainable numbers of younger people to replace the ageing population, the relative size of the population segment aged 65 years old or over will continue to increase. Improved economic circumstances have been exacerbating this impact because of increased welfare and life satisfaction [8].[7]. Other studies identify factors, such as education [9][8] and genetic traits [10][9] that may also be contributing factors impacting population ageing.

The evolution of the nursing workforce into an independent health care profession is evident as nurses now account for over 50% of the global healthcare industry[11]. Worldwide, nurses have and will continue to play over 50% of the global healthcare industry [10]. Worldwide, nurses have and will continue to play a critical role in health promotion, disease prevention and delivery of a comprehensive range of healthcare services [11].[10]. Within primary, secondary and tertiary healthcare settings, the scope of nursing healthcare has afforded nurses' opportunities to lead and coordinate patient care provided by multidisciplinary health professionals. Therefore, the quality of healthcare from nurses should be considered a core indicator of a populations level of healthcare. Previous studies, for example, have revealed that nursing workforce plays a significant role in reducing infant and neonatal mortalities [12] and life expectancy at birth and at 65 years old [13, 14].[11] and life expectancy at birth and at 65 years old [12, 13]. An outcome from the role of nursing workforce in promoting population health has been an increase in the number of who survive past 65 years old.

Ironically, as the population of many countries pushespushing past 65yo, so increase, these countries have the burdens of an ageing population started to further impact available healthcare systems. In recent years the level of demand has grown, at a much faster pace [15].[14]. This impact has contributed to the global nursing staff shortage crisis because old people have higher healthcare dependency on healthcare professionals, especially nursing staff. Studies in individual healthcare facility settings or at community levels have reported that nursing shortages are a chronic issue [16][15], and this was highlighted during the COVID-19 pandemic period [17].[16].

The challenge for health authorities is to take strategic actions to provide sufficient healthcare to an older population with high demand of nursing care. This study helps to illustrate and quantify the role of nursing workforce in increasing the proportion of people over 65 years old. In this study, we hypothesized that, globally, nursing healthcare services may be a significant contributor for increasing the portion of people to live over 65 years. We tested this hypothesis by examining the statistical relationship between nurse density and the 65yo%, globally and regionally. With reference to previous studies into healthcare outcomes of nursing staff, economic affluence, obesity prevalence, physician density, total fertility rate and urbanization were incorporated as potential confounding factors.

Materials and Methods

Data Sources

The country specific data published by the agencies of the United Nations (UN) were extracted for this ecological study. Country in this study does not necessarily indicate political independence but only refers to the geographic territory or region which reported data on health, demography and economic situation to the World Bank ~~[18].~~[17]. Except for the obesity prevalence rate which was extracted from the World Health Organization ~~[19]~~[18], all other variables (nurse density, population ageing level, GDP PPP, physician density, total fertility rate and urbanization) were downloaded from the World Bank database [~~20~~19].

~~1. The dependent variable, population ageing level indexed with the percentage of population aged 65 and above (65yo%) in 2020 [21]~~

1. The dependent variable, population ageing level indexed with the percentage of population aged 65 and above (65yo%) in 2020 [20]

The DataBank of the World Bank is an analysis and visualisation tool that contains collections of time series data on a variety of topics, including socioeconomics, key health, nutrition and demography statistics. Population is based on the de facto definition of population, which counts all residents regardless of legal residency status or citizenship.

2. The independent variable, nurse density measured with the number of nurses and midwives per 1,000 people [~~22~~21]

To reduce random errors at the time of data collection, we averaged the number of nurses and midwives for the period between 2014 and 2018 in each country for our data analyses.

Empirically and as per recent studies, economic affluence, obesity, physician service access, total fertility rate and urban living have been either positive or negatively associated with people's health affecting their opportunities surviving 65 years old. Therefore, they were included as the potential confounders while we analysed the independent role of nursing workforce in promoting population ageing:

3. Economic affluence, expressed with the gross domestic product (GDP) per capita [~~23~~22]

The economic affluence is specifically indexed as GDP PPP per capita (current international $ for purchasing power parity) in 2014. GDP PPP is included as the index of economic affluence in this study as it has the advantage over GDP per capita because it considers the relative cost of local goods, services

and inflation rates of the country. Therefore, GDP PPP is more associated with the life quality and wellbeing of individuals who live in different countries [~~8~~7, 23, 24~~, 25~~].

~~4. Obesity prevalence rate, measured with the percentage of adult individuals with the body mass index (BMI) equal to or exceeding 30 kg/m^2 in 2014 [19]~~

~~Obesity is a result of metabolic imbalances which increases the risk for obese individuals to develop medical complications and die younger than 65 years old [26].~~

4. Obesity prevalence rate, measured with the percentage of adult individuals with the body mass index (BMI) equal to or exceeding 30 kg/m^2 in 2014 [18]

Obesity is a result of metabolic imbalances which increases the risk for obese individuals to develop medical complications and die younger than 65 years old [25].

5. Physician density, expressed with the number of physicians per 1,000 people ~~[27]~~[26]

Physician healthcare access determines not only the level of primary healthcare services, but also the diagnosis and treatment of patients' health conditions at the secondary and tertiary levels~~ [28]. Therefore, physician and nursing healthcare services may be seen to confound with each other for maintaining and improving people's health to facilitate them to survive 65 years old [28]~~. Therefore, physician and nursing healthcare services may be seen to confound with each other for maintaining and improving people's health to facilitate them to survive 65 years old [27].

~~6. Fertility rate (total), representing the total number of children born to a woman [29]~~

6. Fertility rate (total), representing the total number of children born to a woman [28]

It is reasonable to note that falling fertility rates are one of the major determinants of population aging [~~4-7~~3-6]. Low fertility rates result in smaller youth cohorts, which ~~create~~creates an imbalance in the demographic structure: older age groups expand and become more populous than their younger counterparts [~~30~~29].

7. Urbanization, indexed with the percentage of total population living in urban areas in 2014 ~~[31]~~[30]

Urbanization represents a major demographic shift which is characterised by lifestyle changes, for instance lack of physical activity and poor diet patterns [~~32-34~~31-33]. Studies controversially showed that people living in both metropolitan areas and ~~rule~~rural areas have greater life expectancy and higher percentage to survive 65 years old. However, urban residents experience relatively larger gains in life expectancy than those in rural areas [~~35~~34].

The relevant United Nations agencies offer free online access to data required for the analyses such as in this study. The data can only be identifiable to the level of population. They are not identifiable or re-identifiable to the individual participant, their family or community. Therefore, there is no need to obtain ethical approval or consent during the entire study process.

Data Selection

We extracted the country specific data on independent variable (nurse density), dependent variable (population ageing level) and 5 potential confounders (GDP PPP, obesity prevalence, physician density, total fertility rate and urbanization). For each variable, we extracted the data from all the countries where data were available from the websites of the United Nation agencies.

Following the country list created by the World Bank, we aligned all the 7 variables from the individual countries and obtained a full set of data comprising 215 countries. Each country was treated as an individual study subject in all the data analysis models. However, not all the countries (subjects) have all the information for all the variables. The numbers of countries/ subjects (sample size) included for analysing the correlations to other variables may differ as such.

Data analysis

~~To assess the correlation between the nurse density and the 65yo%, the analysis proceeded in the following 5 models [28, 36-39]:~~

To assess the correlation between the nurse density and the 65yo%, the analysis proceeded in the following 5 models [27] [27, 35-38]:

1) Scatter plots were explored with the raw data in Microsoft Excel® for examining and visualizing the strength, shape and direction of correlation of nurse density to 65yo%. Additionally, the scatter plots graph allowed an examination of data quality, for example, if there are any unexpected gaps in the data and if there are any extreme outlier points.

~~Data for producing the scatter plots are raw data which are not log-transformed. However, to~~To reduce the skewness of our original data for valid statistical analysis results produced in the following data analysis models, all the ~~seven~~7 variables were log-transformed.

2. Bivariate (Pearson's r and nonparametric) correlations were conducted to examine the directions and strengths of the correlations between all the variables.

3. Partial correlation of Pearson's moment-product approach was performed to identify the independent correlation relationships between variables. We alternated each of the 6 variables (nurse density, GDP PPP, obesity, physician density, total fertility rate and urbanization) as the independent predictor when all the other 5 variables are included as the potential confounding factors. And then, we alternated each individual variable as the controlled variable to assess the relationship between 65yo% and each of the 5 variables.

4. Standard multiple linear regression (enter) was performed to describe the correlations between the dependent variable (65yo%) and the predicting variables. To explore if and how much nurse density could statistically explain the individual relationships between nurse density and GDP PPP, obesity, physician density, total fertility rate and urbanization, the enter multiple linear regression was performed to calculate the correlations between nurse density and the confounding variables when nurse density is "added" and "not added" as a predicting variable respectively.

Subsequently, standard multiple linear regression (~~Stepwise~~stepwise) is performed to select the most significant predicting variable(s) for 65yo% when nurse density was “added” and “not added” as a predicting variable respectively.

5. The universal correlations between nurse density and 65yo% were explored and compared in different country groupings:

1) the World Bank income classifications: high income, upper middle income, low-middle income and low income;

2) the UN common practice on defining the developed and developing countries ~~[40];~~[39];

Fisher’s r-to-z transformation was applied to compare the nurse density- 65yo% correlations in the developed countries and the developing countries~~;~~.

3) the WHO regional classifications: Africa (AFR), Americas (AMR), Eastern Mediterranean (EMR), Europe (EU), South-East Asia (SEAR) and Western Pacific (WPR) ~~[41];~~[40];

4) countries with the strong contrast in terms of geographic distributions, per capita GDP levels and/or cultural backgrounds. We analysed the correlation in the ~~six~~6 country groupings: Asia Cooperation Dialogue (ACD) ~~[42]~~[41]; the Asia-Pacific Economic Cooperation (APEC); the Arab World [4~~3~~2], European Economic Area (EEA) ~~[44]~~[43], countries with English as the official language (government websites), European Union (EU) ~~[45]~~[44], Latin America ~~[46]~~[45], Latin America and the Caribbean (LAC) ~~[46]~~[45], the Organisation for Economic Co-operation and Development (OECD) and non-OECD group [~~47~~46].

Bivariate correlations, partial correlation, multiple linear regression analyses (enter and stepwise) ~~are~~were conducted with SPSS v. 28. The significance is kept at the 0.05 level, but 0.01 and 0.001 levels ae also reported. Standard multiple linear regression analysis criteria are set at probability of F to enter ≤ 0.05 and probability of F to remove ≥ 0.10.

Results

The relationship identified in the scatterplots between nurse density and population ageing was a strong correlation (R^2= 0.5268 (r= 0.7258), p<0.001, n= 177, Figure 1). The nursing workforce explains 52.68% of population ageing variance.

Figure 1 The relationship between nurse density and population ageing level

This strong relationship between nurse density and population ageing identified in the scatterplots was confirmed by the subsequent bivariate correlation analyses with the log-transformed data.

Globally, nurse density significantly correlated to 65yo% (r= 0.694 and rho= 0.724, p<0.001 respectively in Pearson and non-parametric analyses, Table 1).

Table 1 Pearson (above diagonal) & non-parametric (below diagonal) correlation matrix for all variables

	Nurse density	Population ageing	GDP PPP	Obesity prevalence	Physician density	Total fertility rate	Urbanization
Nurse density	1	0.694***	0.774***	0.508***	0.784***	-0.735***	0.547***
Population ageing	0.724***	1	0.671***	0.365***	0.759***	-0.852***	0.494***
GDP PPP	0.797***	0.686***	1	0.502***	0.839***	-0.799***	0.720***
Obesity prevalence	0.465***	0.365***	0.483***	1	0.500***	-0.391***	0.546***
Physician density	0.810***	0.810***	0.831***	0.445***	1	-0.841***	0.626***
Total fertility rate	-0.745***	-0.872***	-0.777***	-0.352***	-0.816***	1	-0.521***
Urbanization	0.600***	0.506***	0.757***	0.584***	0.630***	-0.522***	1

Significance level: ***p< 0.001; n ranges between 176 and 212.

Data source and definition: Nurse density, expressed with the number of nurses and midwives per 1,000 population (the Word Bank); Population ageing, presented with the percent of population aged 65 and above (the Word Bank); GDP PPP, the per capita purchasing power parity (PPP) value of all final goods and services produced within a territory in a given year (the Word Bank); Obesity prevalence, the percentage of population with BMI ≥30 prevalence (WHO Global Health Observatory); Physician availability, the number of nurses and midwives per 1,000 population (the Word Bank); Total fertility rate, representing the number of children that are born to a woman, the World Bank; Urbanization, the percentage of population living in urban area (the Word Bank);

All the data were log-transformed for correlation analysis.

When predicting and confounding variables (nurse density, GDP PPP, obesity, total fertility rate, physician density and urbanization) were individually correlated to population ageing, while keeping the other 5 variables statistically constant, nurse density, GDP PPP and total fertility rate remained significant correlations to 65yo% (r= 0.194, p< 0.05; r= 0.223, p< 0.01 and r= 0.609, p < 0.001 respectively, Table 2-1). Neither obesity prevalence nor urbanization showed an independent correlation to 65yo% while the other 5 variables were kept statistically constant (Table 2-1).

Moreover, nurse density was in constant and significant correlation to 65yo% when GDP PPP, obesity prevalence, total fertility rate, physician density and urbanization were individually controlled (r = 0.371, p< 0.001; r= 0.634, p< 0.001; r= 0.243, p< 0.001; r= 0.189, p< 0.01 and r= 0.582, p< 0.001 respectively, Table 2-2).

These partial correlation analysis results suggest that, statistically, the individual, or the combined confounding effects of GDP PPP, obesity prevalence, total fertility rate, physician density and urbanization did not affect the significant relationship of nurse density to people increasing over 65yo%.

Table 2 Partial correlation coefficients between Population ageing and nurse density with individual and different combinations of controlled variables

	Table 2-1: Nurse density, GDP PPP, obesity prevalence, physician density, total fertility rate and urbanization were alternated as the predicting variable for calculating its relationship with population ageing level while the other 4 variables were kept statistically constant.											
	Population ageing (65yo%)		Population ageing (65yo%)		Population ageing (65yo%)		Population ageing (65yo%)		Population ageing (65yo%)		Population ageing (65yo%)	
Variables	r	p	r	p	r	p	r	p	r	p	r	p
Nurse density	0.194	<0.050	-	-	-	-	-	-	-	-	-	-
GDP PPP	-	-	-0.223	<0.010	-	-	-	-	-	-	-	-
Obesity prevalence	-	-	-	-	-0.038	0.626	-	-	-	-	-	-
Physician density	-	-	-	-	-	-	0.119	0.120	-	-	-	-
Total fertility rate	-	-	-	-	-	-	-	-	-0.609	< 0.001	-	-
Urbanization	-	-	-	-	-	-	-	-	-	-	0.147	0.055

	Table 2-2: Nurse density, GDP PPP, obesity prevalence, physician density, total fertility rate and urbanization were alternated as the individual potential confounder for exploring the partial correlations between population ageing level and the other 4 independent/confounding variables.											
	Population ageing (65yo%)		Population ageing (65yo%)		Population ageing (65yo%)		Population ageing (65yo%)		Population ageing (65yo%)		Population ageing (65yo%)	
Variables	r	p	r	p	r	p	r	p	r	p	r	p
Nurse density	-	-	0.371	< 0.001	0.634	< 0.001	0.243	< 0.001	0.189	< 0.010	0.582	< 0.001
GDP PPP	0.295	< 0.001	-	-	0.606	< 0.001	0.097	0.196	-0.031	0.683	0.523	< 0.001
Obesity prevalence	0.020	0.793	0.043	0.569	-	-	-0.027	0.720	0.064	0.394	0.130	0.084
Physician density	0.482	< 0.001	0.487	< 0.001	0.716	< 0.001	-	-	0.150	0.043	0.664	< 0.001
Total fertility rate	-0.701	< 0.001	-0.709	< 0.001	-0.828	< 0.001	-0.607	< 0.001	-	-	- 0.802	< 0.001
Urbanization	0.190	< 0.010	0.021	0.782	0.378	< 0.001	0.036	0.629	0.111	0.129	-	-

- Controlled variable; Table 2-1 df = 169; Table 2-2 df range: 173 to 188

Data source and definition Data source and definition: Nurse density, expressed with the number of nurses and midwives per 1,000 population (the Word Bank); Population ageing, presented with the percent of population aged 65 and above (the Word Bank); GDP PPP, the per capita purchasing power parity (PPP) value of all final goods and services produced within a territory in a given year (the Word Bank); Obesity prevalence, the percentage of population with BMI ≥30 prevalence (WHO Global Health Observatory); Physician availability, the number of nurses and midwives per 1,000 population (the Word Bank); Total fertility rate, representing the number of children that are born to a woman, the World Bank; Urbanization, the percentage of population living in urban area (the Word Bank);

All the data were log-transformed for correlation analysis.

Standard multiple linear regression (enter) analysis was applied to further predict 65yo% when nurse density, GDP PPP, obesity, physician density and urbanization were considered as the predicting variables. When nurse density was "not added" as one of the predicting variables, physician density and total fertility rate were in significant correlations to 65yo% (β= 0.230, p<0.01 and β= -0.770, p<0.001 respectively). GDP PPP, obesity prevalence and urbanization showed weak and insignificant correlations to 65yo% (Table 3-1). When nurse density was "added" as a predicting variable, it showed its significant contribution to 65yo% together with total fertility rate (β= 0.178, p<0.010; β= -0.239, p<0.010; β= -0.749, p< 0.001, Table 3-1). Obesity prevalence, physician density and urbanization showed very weak and insignificant relationship to 65yo% (Table 3-1).

Similarly, in the subsequent stepwise linear regression model, when nurse density was "not added" as one of the predictors, total fertility rate and physician density were selected as the two most significant variables which totally explained 72.9% (R^2 = 0.729, Table 3-2) of 65yo% variance. While nurse density was "added" as a predicting variable, the stepwise linear regression model selected nurse density as the 2nd most influential predictor for 65yo% with (R^2 increment= 0.010, Table 3-2). In the stepwise regression analysis model, totally, 74.1% (R^2= 0.741) of 65yo% variance was explained by the 4 selected significant predicting and potential confounding variables (total fertility rate, nurse density, GDPP PPP and physician density, Table 3-2).

Table 3 Multiple linear regression results to show predicting effects of independent variables and identify the significant predictors of population ageing level

**Enter**				
	Population ageing (65yo%)			
	Nurse density (not added)		Nurse density (added)	
Variable	Beta	Significance	Beta	Significance
Nurse density	Not added		0.178	< 0.050
GDP PPP	-0.105	0.065	-0.239	< 0.010
Obesity prevalence	0.029	0.563	0.008	0.874
Physician density	0.230	< 0.010	0.166	0.069
Total fertility rate	-0.770	< 0.001	-0.749	< 0.001
Urbanization	0.011	0.857	0.032	0.607

**Stepwise**					
Population ageing (65yo%)					
Nurse density (not added)			Nurse density (added)		
Model	Variable	Adjusted R^2	Model	Variable	Adjusted R^2
1	Total fertility rate	0.722	1	Total fertility rate	0.722
2	Physician density	0.729	2	Nurse density	0.732
	GDP PPP	Insignificant	3	GDP PPP	0.736
	Obesity prevalence	Insignificant	4	Physician density	0.741
	Urbanization	Insignificant		Obesity prevalence	Insignificant
	Nurse density	Not added		Urbanization	Insignificant

Significance level: * p<0.05; ** p< 0.01; ***p< 0.001

Data source and definition Data source and definition: Nurse density, expressed with the number of nurses and midwives per 1,000 population (the Word Bank); Population ageing, presented with the percent of population aged 65 and above (the Word Bank); GDP PPP, the per capita purchasing power parity (PPP) value of all final goods and services produced within a territory in a given year (the Word Bank); Obesity prevalence, the percentage of population with BMI ≥30 prevalence (WHO Global Health Observatory); Physician availability, the number of nurses and midwives per 1,000 population (the Word Bank); Total fertility rate, representing the number of children that are born to a woman, the World Bank; Urbanization, the percentage of population living in urban area (the Word Bank);

All the data were log-transformed for correlation analysis.

Table 4 showed the relationship between nurse density and 65yo% in different country groupings. The best fit equations and R square extracted from the scatt plots consisting of nurse density and 65yo% showed that nurse density universally correlated to 65yo% in all country groupings. The highlights of the results were the relationships between nurse density and 65yo% in United Nations developed and developing countries (R^2= 0.722 and 0.248 respectively, Table 4). Fisher's r to z transformation revealed that nurse density had the significantly more important role in promoting 65yo% in developed countries than in the developing countries (z= 4.02, p< 0.001, Table 4).

Table 4 Bivariate correlations between Nurse density and population ageing level within various country groupings

Country grouping	Best fit equation	R^2	Trendline pattern	Fisher's r-to-z transformation
UN common practice				
Developed	$y = -0.4141x^2 + 1.7808x + 1.0889$	$R^2 = 0.722$, n=45	Polynomial	Developed vs developing countries: z= 4.02, p< 0.001
Developing	$y = 0.0167x^2 + 0.0891x + 0.6085$	$R^2 = 0.248$, n=141	Polynomial	
World Bank income classifications				
Low	$y = 0.0814x^2 - 0.6136x + 2.1834$	$R^2 = 0.2908$	Polynomial	
Low middle	$y = -0.0249x^2 + 0.5036x - 0.4412$	$R^2 = 0.1924$	Polynomial	
Upper middle	$y = 0.0381x^2 - 0.1774x + 1.7187$	$R^2 = 0.1819$	Polynomial	
High	$y = -0.1445x^2 + 2.5845x - 8.3844$	$R^2 = 0.1590$	Polynomial	
WHO Regions				
AFRO	$y = 0.1026x^2 - 0.815x + 2.6237$	$R^2 = 0.4013$	Polynomial	
AMRO	$y = 0.0176x^2 + 0.0354x + 1.3525$	$R^2 = 0.4841$	Polynomial	
EMRO	$y = -0.167x^2 + 1.6365x - 2.4014$	$R^2 = 0.3026$	Polynomial	
EURO	$y = -0.1331x^2 + 0.8006x + 1.6858$	$R^2 = 0.0552$	Polynomial	
SEARO	$y = -0.1309x^2 + 1.4553x - 2.083$	$R^2 = 0.1015$	Polynomial	
WPRO	$y = 0.18x^2 - 1.634x + 5.0363$	$R^2 = 0.6539$	Polynomial	
Countries grouped based on various factors				
ACD	$y = 0.6558\ln(x) + 0.583$	$R^2 = 0.0359$	Logarithmic	
APEC	$y = 0.6242e^{0.2021x}$	$R^2 = 0.6200$	Exponential	
Arab World	$y = -0.143x^2 + 1.408x - 1.93$	$R^2 = 0.2206$	Polynomial	
EEA	$y = -0.2023x^2 + 0.957x + 1.8014$	$R^2 = 0.3603$	Polynomial	
EOL	$y = 0.1063x^2 - 0.7988x + 2.4714$	$R^2 = 0.5424$	Polynomial	
EU	$y = -0.1635x^2 + 0.6147x + 2.4294$	$R^2 = 0.0776$	Polynomial	
LA	$y = 0.0263x^2 - 0.0409x + 1.4964$	$R^2 = 0.6468$	Polynomial	
LAC	$y = 0.0031x^2 + 0.1594x + 1.1142$	$R^2 = 0.4057$	Polynomial	
OECD	$y = -0.2023x^2 + 0.957x + 1.8014$	$R^2 = 0.3603$	Polynomial	
SADC	$y = 0.1063x^2 - 0.7988x + 2.4714$	$R^2 = 0.5424$	Polynomial	

Data source and definition: Nurse density, measured with the number of nurses and midwives per 1,000 population (the Word Bank); Population ageing, presented with the percent of population aged 65 and above (the Word Bank).

All the data were log-transformed for correlation analysis.

Discussion

This ecological study examined the correlation between nursing density and population ageing (65yo%) with consideration of the confounding effects of economic affluence (GDP PPP), obesity prevalence,

physician density, total fertility rate and urbanization. The findings in this study reveal that nurse density may be a significant contributor to population ageing, and this contributing effect remains independent of economic affluence (GDP PPP), total fertility rate, obesity, physician density and urbanization.

It is well-known that, as the biggest healthcare professional group [11]It is well-known that, as the biggest healthcare professional group [10], nurses are essential to primary, secondary and tertiary health care. This is evident in the intrinsic correlation between nurse density -and improvements to life expectancies at birth and 65 years of age, old within OECD countries [13][13] and worldwide [14].[12].

The impact of nursing has been seen in many domains of health such as management of infectious diseases, a significant contributor to reduced life expectancy. As healthcare professionals in constant communication with patients and their needs nurses have led and manage infection control services across acute and community settings, effectively interrupting disease transmission on multiple levels of population and social status [47, 48, 49]. As the frontline healthcare professional, nurses have been playing a crucial role in preventing COVID- 19 from spreading and treating COVID-19 patients [50, 51].[49, 50]. Nursing interventions have been reducing infectious disease mortality rates across the whole population, including the segment aged 65 and over [52].[51].

High child mortality rates and the ongoing impact of an infectious disease pandemic are significant obstacles for people to survive to 65 years of age. Globally, nurses have been playing a pivotal role in reducing child mortality rate through heavy involvement in communicable and non-communicable disease management [1211, 52, 53, 54]. Thompson and Keeling report that over 40 years, improvements in child mortality have occurred through nurses educating and assisting young mothers on how to feed their children and how to maintain hygiene [53].[52].

Patient education and public health promotion are critical components of the nurses' daily job, which may be even more important concerns for the older population because of the second epidemiology transition [55]. This transition, marked by a rapidly growing public health crisis concerning a shift from infectious diseases to the impact of chronic diseases is a consequence of good nursing care. This has been evidenced in the role of the nursing workforce in contributing to life expectancy both at younger age and then older than 65 across epidemics, acute health conditions [13, 14] and a range of degenerative diseases which occur in older populations [13]. In terms of approach, both studies also correlated nurse density to life expectancy at population level, similar [54]. This transition, marked by a rapidly growing public health crisis concerning a shift from infectious diseases to the impact of chronic diseases is a consequence of good nursing care. This has been evidenced in the role of the nursing workforce in contributing to life expectancy both at younger age and then older than 65 across epidemics, acute health conditions [12, 13] and a range of degenerative diseases which occur in older populations [13]. In terms of approach, both studies also correlated nurse density to life expectancy at population level, similar to those revealed in this study.

Nurses are central to full coverage healthcare services, they are often the first and sometimes the only health professionals that patients see [11].[10]. The quality of healthcare received from nurses has been associated with patient safety [56-6155-60], low mortality rate [62-6761-66] and patient outcomes [56, 60, 68-7155, 59, 67-70]. While the breadth of the impact of nurses on people's health remains difficult to fully

quantify, the community recognition of nursing healthcare services occurs through various awards, for example, the International Year of the Nurse and the Midwife ~~[72]~~[71] and the Gallup Roll [~~73~~72].

Strengths and limitations

~~Free~~The greatest strength of this study is free and unrestricted access to the predicting variable (nurse density) and dependent variable (65yo%) which was complimented with data concerning potential confounding variables (GDP PPP, obesity prevalence, total fertility rate and urbanization). The time based serial data enabled both predicting and confounding variables to observe their delayed presentations and impact on 65yo%. This enabled data analysis using several different models, such as scatterplots, bivariate, partial correlation and linear regression. This level of data availability and subsequent multiple data analysis approaches provide a rich source of findings when compared to individual based studies.

Some limitations to be noted:

Firstly, this is an ecological study, and the results are subject to ecological fallacy. The correlations identified in this study are at population level, but they may not necessarily hold true at the individual level.

Secondly, this is a cross sectional study, the relationship between nurse density and 65yo% is only correlational, not causal.

Thirdly, the data included in this study may be crude and have some random errors when the United Nations agencies collected and aggregated data at population level. However, the analysis results are highly repeatable which may be different from individual data based studies.

Implications for Practice

This study delivers a strong message to health authorities worldwide. As populations age 65 and over increase, the prevalence of disability, frailty and chronic diseases also rises [~~15~~14, 73, 74~~, 75~~]. With the increasing percentage of the world population aged 65+, the pressure for nursing care becomes cumulative, essentially exacerbating the challenges of a shortage of nursing staff. This study should encourage healthcare authorities, to consider the implications increase of density when promoting population health.

Conclusions

Nurse density correlates to population ageing globally and regionally, but is more strongly in developed countries. This suggests that nurse density may be a significant predictor for population ageing, and is more prominent in developed countries. Ironically, nursing healthcare has been partially responsible for population ageing which in turn demands more nursing care and accordingly exposes the nursing profession to a worsening shortage of staff. To highlight the profound implications for the role the nursing profession plays especially at a time of global nursing shortage, further study into the effects of long-run elasticity of nurse staffing level on population ageing may be needed. For instance, what percentage of increase of nursing staff would be required to meet every 1% increase of an ageing population.

~~Ethical approval~~

~~The relevant United Nations agencies offer free online access to data required for the analyses such as in this study. The data can only be identifiable to the level of population. They are not identifiable or re-identifiable to the individual participant, their family or community. Therefore, there is no need to obtain ethical approval or consent during the entire study process.~~

Authors' contributions

WY and FD contributed to the study design. Both authors conducted the data analysis and interpreted data analysis results. WY drafted the manuscript with input from FD. Both authors contributed, read and approved the final manuscript.

Conflicts of Interest

The author declares no conflict of interest.

References

1. ~~OCED Data. *Elderly population (indicator)*. 2022 16 May 2022]; Available from: 10.1787/8d805ea1-en~~

~~2.~~ United Nations. Ageing~~. 2022;~~ Official website: United Nations; 2022. Available from: https://www.un.org/en/global-issues/ageing.

~~3. OECD~~2. OCED Data. Demography- Elderly population~~.~~: Organisation for Economic Co-operation and Development; 2022 ~~-~~[November 12 2022~~]~~;]. Available from: https://data.oecd.org/pop/elderly-population.htm.

~~4~~3. Lee~~,~~ R~~.,~~, Mason A, network N, Lee R, Mason A, Amporfu E, et al~~.,~~. Is low fertility really a problem? Population aging, dependency, and consumption. Science~~,~~. 2014~~.~~;346(6206):~~ p.~~ 229-~~234.~~34. doi: https://doi.org/10.1126/science.1250542.

~~5.~~ 4. ESHRE Capri Workshop Group~~, E.C.W.,~~. Fertility and ageing. Human reproduction update~~,~~. 2005~~.~~;11(3):~~ p.~~ 261-~~276.~~76. doi: https://doi.org/10.1093/humupd/dmi006.

~~6~~5. Cheng~~,~~ X~~.,~~, Yang Y, Schwebel DC, Liu Z, Li L, Cheng P, et al~~.,~~. Population ageing and mortality during 1990–2017: a global decomposition analysis. PLoS medicine~~,~~. 2020~~.~~;17(6):~~ p.~~ e1003138. doi: https://doi.org/10.1371/journal.pmed.1003138.

~~7~~6. Harper~~,~~ S~~.,~~. Living longer within ageing societies. Journal of Population Ageing~~,~~. 2019~~.~~;12(2):~~ p.~~ 133-~~136.~~6. doi: https://doi.org/10.1007/s12062-019-09248-4.

~~8~~7. Marmot~~, M.G. and G.D.~~ MG, Smith~~,~~ GD. Why are the Japanese living longer? BMJ: British Medical Journal~~,~~. 1989~~.~~;299(6715):~~ p.~~ 1547. doi: https://doi.org/10.1136/bmj.299.6715.1547.

~~9~~8. Hummer~~, R.A. and E.M.~~ RA, Hernandez~~,~~ EM. The effect of educational attainment on adult mortality in the United States. Population bulletin~~,~~. 2013~~.~~;68(1):~~ p.~~ 1-16. doi: https://www.ncbi.nlm.nih.gov/pmc/articles/PMC4435622/. PubMed Central PMCID: PMC4435622.

~~10~~9. Timmers~~, P.R., et al.,~~ PR, Wilson JF, Joshi PK, Deelen J. Multivariate genomic scan implicates novel loci and haem metabolism in human ageing. Nature communications~~, 2020. 11(1): p. 1-10.~~. 2020;11(1):1-10. doi: https://doi.org/10.1038/s41467-020-17312-3.

~~11~~10. WHO. Nursing and midwifery~~.~~ Internet The World Health Organization; 2020 [January 8 2022~~]~~;]. Available from: https://www.who.int/news-room/fact-sheets/detail/nursing-and-midwifery.

~~12. Amiri, A., et al.,~~11. Amiri A, Vehviläinen-Julkunen K, Solankallio-Vahteri T, Tuomi S. Impact of nurse staffing on reducing infant, neonatal and perinatal mortality rates: Evidence from panel data analysis in 35 OECD countries. International journal of nursing sciences~~, 2020. 7(2): p. 161-169.~~. 2020;7(2):161-9. doi: https://doi.org/10.1016/j.ijnss.2020.02.002.

12. You W, Donnelly F. Although in shortage, nursing workforce is still a significant contributor to life expectancy at birth. Public Health Nursing. 2022. doi: https://doi.org/10.1111/phn.13158.

13. Amiri~~,~~ A~~. and T.~~, Solankallio-Vahteri~~,~~ T. Nurse staffing and life expectancy at birth and at 65 years old: evidence from 35 OECD countries. International journal of nursing sciences~~, 2019. 6(4): p. 362-370.~~. 2019;6(4):362-70. doi: https://doi.org/10.1016/j.ijnss.2019.07.001.

14. ~~You, W. and F. Donnelly, *Although in shortage, nursing workforce is still a significant contributor to life expectancy at birth* Public Health Nursing 2022.~~

~~15.~~ WHO. Ageing and health~~.~~: The World Health Orgnization 2022 ~~-~~[18 December 2022~~]~~;]. Available from: https://www.who.int/news-room/fact-sheets/detail/ageing-and-health.

~~16~~15. Tamata~~, A.T. and M.~~ AT, Mohammadnezhad~~,~~ M. A systematic review study on the factors affecting shortage of nursing workforce in the hospitals. Nursing open~~,~~. 2023~~.~~;10(3):~~ p.~~ 1247-~~1257.~~57. doi: https://doi.org/10.1002/nop2.1434.

~~17~~16. Xu~~,~~ H~~., O.~~, Intrator~~, and J.R.~~ O, Bowblis~~,~~ JR. Shortages of staff in nursing homes during the COVID-19 pandemic: what are the driving factors? Journal of the American Medical Directors Association~~, 2020. 21(10): p. 1371-1377.~~. 2020;21(10):1371-7. doi: https://doi.org/10.1016/j.jamda.2020.08.002.

~~18~~17. The World Bank. How does the World Bank classify countries? : The World Bank; 2022 ~~-~~[December 24 2022~~]~~;]. Available from: https://datahelpdesk.worldbank.org/knowledgebase/articles/378834-how-does-the-world-bank-classify-countries.

~~19~~18. WHO. GHO | By category | Obesity (body mass index ≥ 30),age-standardized (%) - Estimates by country~~. WHO~~: World Health Organization; 2017 [updated MINERVA PUBLISH ~~DATE 28~~DATE28 August 2017~~]~~;]. Available from: http://apps.who.int/gho/data/view.main.CTRY2450A.

~~20~~19. The World Bank. DataBank~~. World Development Indicators (Public) 2022~~ Official website2022 [May 18 2022~~]~~;]. Available from: https://databank.worldbank.org/home.aspx.

~~21~~20. The world Bank. Population ages 65 and above (% of total population~~).~~) Officiel website: The World Bank; 2020 ~~-~~[cited 2022 16 May 2022~~]~~;]. Available from: https://data.worldbank.org/indicator/SP.POP.65UP.TO.ZS.

~~22. The world~~21. World Bank. Nurses and midwives (per 1,000 people~~). 2014~~) DataBank: the World Bank Group; 2022 ~~-~~[January 18 2022~~]~~;]. Available from: https://data.worldbank.org/indicator/SH.MED.NUMW.P3.

~~23. The world~~22. World Bank. GDP per capita, PPP (current international ~~$). 2014~~ $) Internet2019 [December 28 2021~~]~~;]. Available from: https://data.worldbank.org/indicator/NY.GDP.PCAP.PP.CD.

23. ~~24.~~ Becker~~, G.S., T.J.~~ GS, Philipson~~, and R.R.~~ TJ, Soares~~,~~ RR. The quantity and quality of life and the evolution of world inequality. American economic review~~,~~. 2005~~.~~;95(1):~~ p.~~ 277-~~291.~~91. doi: https://doi.org/10.1257/0002828053828563.

~~25.~~ 24. Deaton~~,~~ A~~.,~~. Income, health, and well-being around the world: Evidence from the Gallup World Poll. Journal of Economic perspectives~~,~~. 2008~~.~~;22(2):~~p.~~ 53-72. doi: https://doi.org/10.1257/jep.22.2.53.
~~26. Peeters, A., et al.,~~25. Peeters A, Barendregt JJ, Willekens F, Mackenbach JP, Al Mamun A, Bonneux L. Obesity in adulthood and its consequences for life expectancy: a life-table analysis. Annals of internal medicine~~, 2003. **138**(1): p. 24-32.~~. 2003;138(1):24-32. doi: https://doi.org/10.7326/0003-4819-138-1-200301070-00008.
~~27. The~~ 26. World Bank. Physicians (per 1,000 people~~).~~) Online: The World Bank; 2020 ~~.~~[April 3 2022~~];~~]. Available from: https://data.worldbank.org/indicator/SH.MED.PHYS.ZS.
~~28~~27. You~~,~~ W~~. and F.~~, Donnelly~~,~~ F. Physician care access plays a significant role in extending global and regional life expectancy. European Journal of Internal Medicine~~, 2022.~~. 2022;(103):62-8. doi: https://doi.org/10.1016/j.ejim.2022.06.006.
~~29~~28. The world Bank. Fertility rate, total (births per woman~~).~~) Official website: The World Bank; 2015 ~~.~~[16 May 2022~~];~~]. Available from: https://data.worldbank.org/indicator/SP.DYN.TFRT.IN.
29. ~~30.~~ Guseh~~, J.S.,~~ JS. Aging of the World's Population. Encyclopedia of Family Studies~~,~~. 2016:~~p.~~ 1-5. doi: https://doi.org/10.1002/9781119085621.wbefs352.
30. ~~31. The~~ World Bank. Urban population (% of total population~~). 2014~~) Official Website: The World Bank Group; 2022 ~~.~~[January 18 2022~~];~~]. Available from: https://data.worldbank.org/indicator/SP.URB.TOTL.IN.ZS.
~~32~~31. Allender~~,~~ S~~., et al.,~~, Foster C, Hutchinson L, Arambepola C. Quantification of urbanization in relation to chronic diseases in developing countries: a systematic review. ~~J Urban Health, 2008. **85**(6): p. 938-951.~~Journal of urban health : bulletin of the New York Academy of Medicine. 2008;85(6):938-51. Epub 2008/10/22. doi: https://doi.org/10.1007/s11524-008-9325-4.
32. ~~33.~~ Moore~~,~~ M~~., P.~~, Gould~~, and B.S.~~ P, Keary~~,~~ BS. Global urbanization and impact on health. International journal of hygiene and environmental health~~,~~. 2003~~.~~;206(4):~~p.~~ 269-~~278.~~78. doi: https://doi.org/10.1078/1438-4639-00223.
~~34.~~ 33. WHO. Urban health~~.~~ WHO Official Website: World Health Organization; 2022 [March 12 2022~~];~~]. Available from: https://www.who.int/health-topics/urban-health#tab=tab_1.
~~35~~34. Singh~~, G.K. and M.~~ GK, Siahpush~~,~~ M. Widening rural–urban disparities in life expectancy, US, 1969–2009. American journal of preventive medicine~~,~~. 2014~~.~~;46(2):~~p.~~ e19-e29. doi: https://doi.org/10.1016/j.amepre.2013.10.017.
~~36~~35. You~~,~~ W~~., et al.,~~, Henneberg R, Coventry BJ, Henneberg M. Cutaneous malignant melanoma incidence is strongly associated with European depigmented skin type regardless of ambient ultraviolet radiation levels: evidence from Worldwide population-based data. AIMS Public Health~~, 2022. **9**(2): p. 378.~~. 2022;9(2):378. doi: https://doi.org/10.3934/publichealth.2022026.
~~37~~36. You~~,~~ W~~., R.~~, Henneberg~~, and M.~~ R, Henneberg~~,~~ M. Healthcare services relaxing natural selection may contribute to increase of dementia incidence. Scientific Reports~~, 2022. **12**(1): p. 1-10.~~. 2022;12(1):1-10. doi: https://doi.org/10.1038/s41598-022-12678-4.
37. ~~38.~~ You~~,~~ W~~., et al.,~~, Rühli F, Eppenberger P, Galassi FM, Diao P, Henneberg M. Gluten consumption may contribute to worldwide obesity prevalence. Anthropological Review~~,~~. 2020~~.~~;83(3):~~p.~~ 327-~~348.~~48. doi: http://dx.doi.org/10.2478/anre-2020-0023.
~~39.~~ 38. You~~,~~ W~~. and M.~~, Henneberg~~,~~ M. Large household reduces dementia mortality: A cross-sectional data analysis of 183 populations. PloS one~~,~~. 2022~~.~~;17(3):~~p.~~ e0263309. doi: https://doi.org/10.1371/journal.pone.0263309.

4039. United Nations Statistics Division. Composition of macro geographical (continental) regions, geographical sub-regions, and selected economic and other groupings.: United Nations; 2013 -[03.10.2016];2022]. Available from: http://unstats.un.org
40. 41. WHO. WHO regional offices.: The World Health Orgnization 2018 11.[November 26.2015];2022]. Available from: http://www.who.int.
41. 42. Asia Cooperation Dialogue. Member Countries. 2018;. Available from: http://www.acddialogue.com.
43. 42. The World Bank. Arab World | Data.: The World Bank; 2015;. Available from: http://data.worldbank.org/region/ARB.
43. 44. European Free Trade Association. What is the European Economic Area? 2016;2022. Available from: http://www.efta.int/eea.
45. 44. European Union. EUROPA - Countries. 2022 -[18.06.2015];]. Available from: http://europa.eu.
4645. The United Nations Educational Scientific and Cultural Organization. UNESCO Regions-Latin America and the Caribbean. 2014 [2022 [[11.26.2015];]. Available from: http://www.unesco.org.
46. 47. OECD. List of OECD Member countries. 2015;. Available from: http://www.oecd.org.
48. 47. You, W., et al.,You W, Galassi FM, Varotto E, Henneberg M. Genghis Khan's death (AD 1227): An unsolvable riddle or simply a pandemic disease? International Journal of Infectious Diseases, 2021. **104**: p. 347-348.. 2021;104:347-8. doi: https://doi.org/10.1016/j.ijid.2020.12.089.
49. Lam, S.K.K., et al.,48. Lam SKK, Kwong EWY, Hung MSY, Chien WT. Emergency nurses' perceptions regarding the risks appraisal of the threat of the emerging infectious disease situation in emergency departments. International journal of qualitative studies on health and well-being, 2020. **15**(1): p. 1718468.. 2020;15(1):1718468. doi: https://doi.org/10.1080/17482631.2020.1718468.
49. 50. Al Thobaity, A. and F., Alshammari, F. Nurses on the frontline against the COVID-19 pandemic: an integrative review. Dubai Medical Journal,. 2020.;3(3): p. 87-92. doi: https://doi.org/10.1159/000509361.
51. 50. Fawaz, M., H., Anshasi, and A. H, Samaha, A. Nurses at the front line of COVID-19: Roles, responsibilities, risks, and rights. The American journal of tropical medicine and hygiene,. 2020.;103(4): p. 1341. doi: https://doi.org/10.4269/ajtmh.20-0650.
5251. Stirling, B., P., Littlejohn, and M.L. P, Willbond, ML. Nurses and the Control of Infectious Disease. Understanding epidemiology and disease transmission is vital to nursing care. Canadian Nurse,. 2004.;100(9): p. 16-20. PubMed Central PMCID: PMC15623008.
5352. Thompson, M.E. and A.A. ME, Keeling, AA. Nurses' Role in the Prevention of Infant Mortality in 1884–1925: Health Disparities Then and Now. Journal of pediatric nursing, 2012. **27**(5): p. 471-478.. 2012;27(5):471-8. doi: https://doi.org/10.1016/j.pedn.2011.05.011.
5453. Amiri, A.,. Role of nurses and midwives in improving patient safety during childbirth: evidence from obstetric trauma in OECD countries. Applied Nursing Research, 2020. **56**: p. 151343.. 2020;56:151343. doi: https://doi.org/10.1016/j.apnr.2020.151343.
5554. McKeown, R.E., RE. The Epidemiologic Transition: Changing Patterns of Mortality and Population Dynamics. Am J Lifestyle Med, 2009. **3**(1 Suppl): p. 19S-26S. 2009;3(1 Suppl):19S-26S. Epub 2010/02/18. doi: https://doi.org/10.1177/1559827609335350. PubMed PMID: 20161566; PubMed Central PMCID: PMC2805833.

5655. Aiken, L.H., LH, Sermeus W, Van den Heede K, Sloane DM, Busse R, McKee M, et al.,. Patient safety, satisfaction, and quality of hospital care: cross sectional surveys of nurses and patients in 12 countries in Europe and the United States. Bmj, 2012. 344.BMJ. 2012;344(e1717). doi: https://doi.org/10.1136/bmj.e1717.
5756. Hughes, R.G., RG. Chapter 44 Tools and strategies for quality improvement and patient safety. Patient safety and quality: An evidence-based handbook for nurses, 2008. Rockville (MD): Agency for Healthcare Research and Quality (US). 2008. doi: https://www.ncbi.nlm.nih.gov/books/NBK2682/.
5857. Wu, Y.,, Fujita S, Seto K, Ito S, Matsumoto K, Huang C-C, et al.,. The impact of nurse working hours on patient safety culture: a cross-national survey including Japan, the United States and Chinese Taiwan using the Hospital Survey on Patient Safety Culture. BMC health services research, 2013. 13(1): p. 1-7.. 2013;13(1):1-7. doi: https://doi.org/10.1186/1472-6963-13-394.
5958. Ausserhofer, D.,, Zander B, Busse R, Schubert M, De Geest S, Rafferty AM, et al.,. Prevalence, patterns and predictors of nursing care left undone in European hospitals: results from the multicountry cross-sectional RN4CAST study. BMJ quality & safety, 2014. 23(2): p. 126-135.. 2014;23(2):126-35. doi: https://doi.org/10.1136/bmjqs-2013-002318.
6059. Cho, E.,, Lee N-J, Kim E-Y, Kim S, Lee K, Park K-O, et al.,. Nurse staffing level and overtime associated with patient safety, quality of care, and care left undone in hospitals: a cross-sectional study. International journal of nursing studies, 2016. 60: p. 263-271.. 2016;60:263-71. doi: https://doi.org/10.1016/j.ijnurstu.2016.05.009.
6160. Amiri, A., T., Solankallio-Vahteri, and S. T, Tuomi, S. Role of nurses in improving patient safety: evidence from surgical complications in 21 countries. International journal of nursing sciences, 2019. 6(3): p. 239-246.. 2019;6(3):239-46. doi: https://doi.org/10.1016/j.ijnss.2019.05.003.
61. 62. Aiken, L.H., et al., LH, Clarke SP, Sloane DM, Sochalski J, Silber JH. Hospital nurse staffing and patient mortality, nurse burnout, and job dissatisfaction. Jama,. 2002.;288(16): p. 1987-1993.93. doi: https://doi.org/10.1001/jama.288.16.1987.
63. 62. Aiken, L.H., et al.,Aiken LH, Cimiotti JP, Sloane DM, Smith HL, Flynn L, Neff DF. Effects of nurse staffing and nurse education on patient deaths in hospitals with different nurse work environments. The Journal of nursing administration,. 2012.;42(10 Suppl): p. S10. doi: https://doi.org/10.1097/MLR.0b013e3182330b6e.
63. 64. Estabrooks, C.A., et al., CA, Midodzi WK, Cummings GG, Ricker KL, Giovannetti P. The impact of hospital nursing characteristics on 30-day mortality. Nursing research,. 2005.;54(2): p. 74-84. doi: https://doi.org/10.1097/00006199-200503000-00002.
65. 64. Van den Heede, K., et al., Lesaffre E, Diya L, Vleugels A, Clarke SP, Aiken LH, et al. The relationship between inpatient cardiac surgery mortality and nurse numbers and educational level: analysis of administrative data. International journal of nursing studies, 2009. 46(6): p. 796-803.. 2009;46(6):796-803. doi: https://doi.org/10.1016/j.ijnurstu.2008.12.018.
6665. Cho, E.,, Sloane DM, Kim E-Y, Kim S, Choi M, Yoo IY, et al.,. Effects of nurse staffing, work environments, and education on patient mortality: an observational study. International journal of nursing studies, 2015. 52(2): p. 535-542.. 2015;52(2):535-42. doi: https://doi.org/10.1016/j.ijnurstu.2014.08.006.
6766. Manojlovich, M.,. Increasing nurse staffing levels and a higher proportion with bachelor's degrees could decrease patient mortality risk. Evidence-based nursing, 2015. 18(2): p. 62-62.. 2015;18(2):62-. doi: https://doi.org/10.1136/eb-2014-101913.

67. ~~68.~~ Oliver~~, G.M., et al.,~~ GM, Pennington L, Revelle S, Rantz M. Impact of nurse practitioners on health outcomes of Medicare and Medicaid patients. Nursing Outlook~~,~~. 2014~~.~~ ;62(6):~~ p.~~ 440-~~447.~~7. doi: https://doi.org/10.1016/j.outlook.2014.07.004.

68. ~~69.~~ Aiken~~, L.H., et al.,~~ LH, Cerón C, Simonetti M, Lake ET, Galiano A, Garbarini A, et al. Hospital nurse staffing and patient outcomes. Revista Médica Clínica Las Condes~~,~~. 2018~~.~~ ;29(3):~~ p.~~ 322-~~327.~~7. doi: https://doi.org/10.1016/j.rmclc.2018.04.011.

69. ~~70.~~ Rafferty~~, A.~~ AM, Clarke SP, Coles J, Ball J, James P, McKee M~~.~~, et al~~.~~. Outcomes of variation in hospital nurse staffing in English hospitals: cross-sectional analysis of survey data and discharge records. International journal of nursing studies~~, 2007. **44**(2): p. 175-182.~~. 2007;44(2):175-82. doi: https://doi.org/10.1016/j.ijnurstu.2006.08.003.

~~71~~70. Suhonen~~,~~ R~~.~~, Papastavrou E, Efstathiou G, Tsangari H, Jarosova D, Leino-Kilpi H, et al~~.~~. Patient satisfaction as an outcome of individualised nursing care. Scandinavian journal of caring sciences~~, 2012. **26**(2): p. 372-380.~~. 2012;26(2):372-80. doi: https://doi.org/10.1111/j.1471-6712.2011.00943.x.

~~72~~71. WHO. Year of the Nurse and the Midwife 2020 ~~-~~[28 June 2022~~]~~;]. Available from: https://www.who.int/campaigns/annual-theme/year-of-the-nurse-and-the-midwife-2020.

~~73~~72. Oakland University. Gallup Poll finds nursing is most honest, ethical profession~~.~~ Internet: Oakland University; 2021~~;~~. Available from: https://oakland.edu/oumagazine/news/nursing/2021/gallup-poll-finds-nursing-is-most-honest-ethical-profession.

~~74~~73. Australian Institute of Health and Welfare. Australian Institute of Health and Welfare Feather Article~~. 6.9 Ageing and the health system: challenges, opportunities and adaptations~~ 2014 ~~-~~[18 December 2022~~]~~;]. Available from: https://www.aihw.gov.au/getmedia/19dbc591-b1ef-4485-80ce-029ff66d6930/6_9-health-ageing.pdf.aspx.

~~75~~74. National Academy of Sciences~~,~~. Retooling for an Aging America: Building the Health Care Workforce~~, in~~. In: Americans. IoMUCotFHCWfO, editor. 2 Health Status and Health Care Service Utilization~~, I.o.M.U.C.o.t.F.H.C.W.f.O. Americans., Editor.~~. Washington (DC): National Academies Press (US); 2008.

PONE-D-22-35617R2
Greater nurse density correlates to higher level of population ageing globally, but is more prominent in developed countries.
PLOS ONE

Journal Requirements:

Please review your reference list to ensure that it is complete and correct. If you have cited papers that have been retracted, please include the rationale for doing so in the manuscript text, or remove these references and replace them with relevant current references. Any changes to the reference list should be mentioned in the rebuttal letter that accompanies your revised manuscript. If you need to cite a retracted article, indicate the article's retracted status in the References list and also include a citation and full reference for the retraction notice.

Authors: We have checked and amended the references. We ensure that:

1) Every reference cited in the text is also present in the reference list (and vice versa)

2) Follow the standard reference style of the PlosOne journal.

3) Each journal articles have the DOI's if the journals have allocated them to the articles.

4) Webpage reference have the title, hyperlink and access data.

We do not need to cite a retracted or unpublished article in this study.

The details about all the amendments on the references can be viewed in the section of References in the revised manuscript with track changes.

---

6. Review Comments to the Author

Reviewer #3: The author has made significant revisions to the suggestions mentioned above, and will further refine the language of the article to avoid ambiguity.

Authors: We have tried to refine the language of the article for improving the clarity and readability.

Does the author have any methods, interventions, and subsequent research to address this topic? Does rationality and necessity exist, and is there any significance for further in-depth research. The author can briefly narrate

Authors: Based on the study finding in this article, we suggested that further studies into the long-run elasticity of nurse staffing level on population ageing may be needed. For instance, what percentage of increase of nursing staff would be required to meet every 1% increase of an ageing population. The expected study area has been added at the end of the Conclusion.
