# Supplementary material for: Greater nurse density correlates to higher level of population ageing globally, but is more prominent in developed countries
Source: PLoS One. 2023 Sep 29;18(9):e0292371. doi: 10.1371/journal.pone.0292371 (PMC10540962; doi:10.1371/journal.pone.0292371)
Supplement: Attachment — Submitted filename: Reviewer Attachments.docx [file pone.0292371.s001.docx]

After analyzing the article, the following changes need to be made.

1. Please check the grammar and writing of the whole text. If the background section，greater nurse density correlates to higher levels of population ageing for those aged 65 or over. Methods：Population level data was extracted for exploring the correlation between nurse density and population 。

2.The results section of the abstract .nursing density has the second greatest influence on population ageing. Can care density affect population aging? Or does the order of words need to be switched.

3. Introduction :We support this hypothesis through calculating the correlation between nursing healthcare level and prevalence of older people (percentage of people aged 65 or above). Is it inconsistent with what is expressed in the title?

4. The r and R of the result section Figure 2-2 need to be unified.

5. Total fertility rate and urbanization show very weak and insignificant correlations to population ageing level (Table 3). I assume it should be total fertility rate and Physician density rather than urbanization.

6. The discussion is too complicated, so we can go straight to the topic and analyze the result part in depth, and many contents of the text are not very relevant.
