# Supplementary material for: Greater nurse density correlates to higher level of population ageing globally, but is more prominent in developed countries
Source: PLoS One. 2023 Sep 29;18(9):e0292371. doi: 10.1371/journal.pone.0292371 (PMC10540962; doi:10.1371/journal.pone.0292371)
Supplement: Attachment — Submitted filename: 0 Response to Review Decision.docx [file pone.0292371.s002.docx]

PONE-D-22-35617

Greater nurse density correlates to higher level of population ageing globally, but is more prominent in developed countries.

PLOS ONE

# Reviewer #1:

1. The study presents the results of original research.

2. Results reported have not been published elsewhere.

3. Experiments, statistics, and other analyses are performed to a high technical standard and are described in sufficient detail.

4. Conclusions are presented in an appropriate fashion and are supported by the data.

5. The article is presented in an intelligible fashion and is written in standard English.

6. The research meets all applicable standards for the ethics of experimentation and research integrity.

7. The article adheres to appropriate reporting guidelines and community standards for data availability.

Authors: We are pleased to hear that this reviewer appreciates our work.

A large majority of the previous studies into the relationship between nursing workforce and population health area drew a similar conclusion that more nurses are required to improve population health. This study argues that:

1) While nurse density may contribute significantly to population ageing (65yo%) globally.

2) Ironically, populations with higher nurse density have a greater percentage of population segment aged +65 or over, which accumulates healthcare dependency on nursing staff. This association may be enhancing the global issue of nursing staff shortage.

Therefore, the finding in this study may be another alert to the health authorities that nursing staff shortage is worse than previously thought because of the increase of 65yo% in almost all populations.

# Reviewer #2:

Q1: your Introduction part is shallow and needs great attention and modification,

Authors: We outlined the previous studies exploring the causes for population ageing, and incorporated the role of nursing workforce in promoting population health and identified the knowledge gap for justifying this study further:

*Previous studies revealed that nursing workforce played a significant role in reducing infant and neonatal mortalities [1] and life expectancy at birth and at 65 years old [2, 3]. An outcome from the role of nursing workforce in promoting population health may be that it has brought more people to survive 65 years old or ageing population group.*

*In the recent years, it has been a challenge for every country that the size and proportion of older persons in each country are growing and the pace is much faster than ever [4]. This has been exacerbating the global nursing staff shortage crisis because old people have higher healthcare dependency on healthcare professionals, especially nursing staff. Studies in individual healthcare facility settings or community levels have reported that nursing shortage has been a chronic issue [5], and was highlighted during the COVID-19 pandemic period [6].*

Additionally, we also specified the study purpose and how to achieve this purpose in the Background as well:

*To alert the health authorities to take strategic plan to provide sufficient healthcare to the old population with high demand of nursing care, it is worth quantifying the role of nursing workforce in increasing the portion of old population (65yo%). In this study---*

Q2: your method part needs grammer correction and you should devoite your time again on this part,

Authors: We have checked the grammar issue in the Materials and Methods. Also, we indicated the data sources in the first paragraph, and then we only listed the variable with phrases with the reference at the end. This does improve the clarity of this section.

Q2: the reason for your log transformation is not clear

Authors: At the end of the “Materials and Methods”, we briefly mentioned the reason for the “log transformation” of our data:

*The variables are log transformed to increase homoscedasticity for the correlation analyses.*

Now we have moved this sentence and elaborated it before including it after the “Data analysis- Model 1” where we started the data analyses with log-transformed data:

*However, to reduce the skewness of our original data for valid statistical analysis results, all the seven variables were log-transformed.*

We also expanded the purpose for us to conduct scatter plots:

*Additionally, the scatter plots graph allowed us to examine the data quality, for example, if there are any unexpected gaps in the data and if there are any extreme outlier points.*

# Reviewer #3:

After analyzing the article, the following changes need to be made.

1. Please check the grammar and writing of the whole text. If the background section，greater nurse density correlates to higher levels of population ageing for those aged 65 or over. Methods：Population level data was extracted for exploring the correlation between nurse density and population 。

Authors: Sorry for the confusion due to inconsistent expressions for nursing healthcare services and population ageing. Now we have defined these two variables and specified their acronyms (nurse density and 65yo% respectively) at the beginnings of the Abstract and the Introduction. They were indicated in the Section of “Materials and Methods- Data Sources” when we specified the data sources and defined each variable. The two acronyms were consistently included in the whole manuscript which improved the clarity of the manuscript.

We have checked the whole manuscript and amended multiple grammar issues. The senior author of this study has put lots of efforts on the language in this manuscript.

2.The results section of the abstract .nursing density has the second greatest influence on population ageing. Can care density affect population aging? Or does the order of words need to be switched.

Authors: The rationale for this study is that:

A country with high nurse density (the number of nurses per 1,000 people) means that more nursing healthcare services are available to the population. More nursing healthcare services would make more people survive 65 years leading up to higher level of population ageing (measured with the percentage of people aged 65 or over).

Therefore, the logical order should be more nursing healthcare (greater nurse density) led to greater level of population ageing (65yo%).

3. Introduction: We support this hypothesis through calculating the correlation between nursing healthcare level and prevalence of older people (percentage of people aged 65 or above). Is it inconsistent with what is expressed in the title?

Authors: Yes, they are consistent in the title and the text.

The wording, “nurse density” is the same expression in the title and manuscript. The phrase, “level of population ageing” has now been defined in the Abstract- Background, the first paragraph of the Introduction and the dependent variable of the “Materials and Methods- Data Sources”.

4. The r and R of the result section Figure 2-2 need to be unified.

Authors: This has been corrected in Table 2. Thanks.

5. Total fertility rate and urbanization show very weak and insignificant correlations to population ageing level (Table 3). I assume it should be total fertility rate and Physician density rather than urbanization.

Authors: Apologies. There were some typos in Table 3-1 (enter) which have been corrected. The following snips have been reported in Table 3-1 (enter):

| Nursing density (not added) | Nursing density (added) |
| --- | --- |
|  |  |

To facilitate author’s reporting and audience reading, we numbered the results from the enter model as Table 3-1 and from stepwise as Table 3-2.

The reporting of Table 3-1 has been amended and the following paragraph has been rewritten in the Results:

*When nursing healthcare is “not added” as one of the predicting variables, physician density and total fertility rate were in significant correlations to 65yo% (β= 0.230, p<0.01 and β= -0.770, p<0.001 respectively). GDP PPP, obesity and urbanization show weak and insignificant correlations to population ageing level (Table 3-1).*

We also checked the other tables and ensure that the results are reported correctly.

6. The discussion is too complicated, so we can go straight to the topic and analyze the result part in depth, and many contents of the text are not very relevant.

Authors: We have removed the repetitions and merged the role of nursing workforce in promoting the health of different population segments.

Additionally, we also removed the last paragraph from the Discussion and merged it into the existing section, Implications for Practice.

Now the total number of words is 520, which was reduced from original 924.

We also improved the clarity of the Conclusions.

# References:

1.	Amiri, A., et al., *Impact of nurse staffing on reducing infant, neonatal and perinatal mortality rates: Evidence from panel data analysis in 35 OECD countries.* International journal of nursing sciences, 2020. **7**(2): p. 161-169.

2.	Amiri, A. and T. Solankallio-Vahteri, *Nurse staffing and life expectancy at birth and at 65 years old: evidence from 35 OECD countries.* International journal of nursing sciences, 2019. **6**(4): p. 362-370.

3.	You, W. and F. Donnelly, *Although in shortage, nursing workforce is still a significant contributor to life expectancy at birth* Public Health Nursing 2022.

4.	WHO. *Ageing and health*. 2022 18 December 2022]; Available from: <https://www.who.int/news-room/fact-sheets/detail/ageing-and-health>.

5.	Tamata, A.T. and M. Mohammadnezhad, *A systematic review study on the factors affecting shortage of nursing workforce in the hospitals.* Nursing open, 2023. **10**(3): p. 1247-1257.

6.	Xu, H., O. Intrator, and J.R. Bowblis, *Shortages of staff in nursing homes during the COVID-19 pandemic: what are the driving factors?* Journal of the American Medical Directors Association, 2020. **21**(10): p. 1371-1377.
