# Supplementary material for: Greater nurse density correlates to higher level of population ageing globally, but is more prominent in developed countries
Source: PLoS One. 2023 Sep 29;18(9):e0292371. doi: 10.1371/journal.pone.0292371 (PMC10540962; doi:10.1371/journal.pone.0292371)
Supplement: Attachment — Submitted filename: REVIEW PLOS.docx [file pone.0292371.s003.docx]

The discussion section is not profound, can a brief sentence summarize this study?

The model can only be used as a reference. Is there any other literature to support care density effect population aging?

The main results of the conclusion section need to be written out

The conclusion drawn from relevant analysis can only be a hypothesis, but cannot accurately confirm a certain result. Data analysis is somewhat simple. Can we further conduct data analysis and explore at a deeper level.
