# Supplementary material for: Greater nurse density correlates to higher level of population ageing globally, but is more prominent in developed countries
Source: PLoS One. 2023 Sep 29;18(9):e0292371. doi: 10.1371/journal.pone.0292371 (PMC10540962; doi:10.1371/journal.pone.0292371)
Supplement: Attachment — Submitted filename: 0 REVIEW PLOS_FD.docx [file pone.0292371.s004.docx]

1. The discussion section is not profound,

Following the first-round reviewer’s request to simplify the discussion (*The discussion is too complicated, so we can go straight to the topic and analyze the result part in depth, and many contents of the text are not very relevant),* the discussion and hypothesis was made more obvious. We believe the profound nature of the discussion is now more obvious, well established, and supportive of the hypothesis. We explained why nurse density is important for increasing the percentage of the population segment aged 65 or over. We achieved this through describing that nursing workforce provides full coverage healthcare services across primary, secondary, and tertiary levels of healthcare.

We also aligned our study with previously published studies arguing that nurse density promotes ageing [1-3]. This was to give weight to the importance of the paper; this is described in the second paragraph of the discussion.

We suggest the profound nature of the study will come through the supporting literature demonstrating the criticality of the nursing workforce to many sectors of health care. For example within OECD (Organisation for Economic Co-operation and Development), nurse density has been also associated with patient safety during childbirth [4], reducing infant, neonatal and perinatal mortality rates [5], alleviating health expenditure as the economic stimulus [6], patient safety within the settings of surgical complications [7] and quality of acute care services [8]:

The role of nurse density has been associated with population health, especially in reducing under 5 mortality is critically important for promoting population ageing.

2. can a brief sentence summarize this study?

A brief sentence to summarize this study has been in the manuscript which is the second sentence of the first paragraph of the Discussion:

*The findings in this study reveal that nurse density may be a significant contributor to population ageing, and this contributing effect remains independent of economic affluence (GDP PPP), total fertility rate, obesity, physician density and urbanization.*

3. The model can only be used as a reference.

We agree that modelling in such studies is referential, circumstantial and possibly controversial. Therefore, they are just references. However, each study contributes new knowledge, generates debate, and offers new insights for further investigation. Such insights are important for planners and health authorities to understand the critical position nurses play in improving population health. This is all important when considering the global nursing staff shortage issue.

The above has been detailed in the section of “Implications for Practice”. Also, we added a sentence at the end of the section of “Implications for Practice” highlight that the founding in this study is just a reference.

*However, this study just serves as one of the references for healthcare authorities, and more studies may be required before health authorities consider an increase of density for promoting population health.*

4. Is there any other literature to support care density effect population aging?

As mentioned in our answer to Question 1, there are two studies to support that care density correlates with population ageing. These two studies are:

1. Amiri, A. and T. *Solankallio*-Vahteri, *Nurse staffing and life expectancy at birth and at 65 years old: evidence from 35 OECD countries.* International journal of nursing sciences, 2019. **6**(4): p. 362-370.

2. You, W. and F. Donnelly, *Although in shortage, nursing workforce is still a significant contributor to life expectancy at birth.* Public Health Nursing 2022. <https://doi.org/10.1111/phn.13158>

We have already cited them to support our hypothesis. Now, we have highlighted that they adopted the similar study approach to ours in this study. The sentence below has been added at the end of the fifth paragraph of the Discussion:

*In terms of study approach, both studies also correlated nurse density to life expectancy at population level, which is very similar to those adopted in this study.*

5. The main results of the conclusion section need to be written out

Authors: The Conclusion in the main text has been revised, which is described at three levels:

1. Statistical study findings: *Nurse density correlates to population ageing globally and regionally, but is more strongly in developed countries.*

2. Suggestion of statistical study finding: *This suggests that nurse density may be a significant predictor for population ageing, and it more prominent in developed countries.*

3. Significance of the study finds for the healthcare management: *Ironically, nursing healthcare has been partially responsible for population ageing which in turn demands more nursing care and accordingly exposes the nursing profession to a worsening shortage of staff.*

6. The conclusion drawn from relevant analysis can only be a hypothesis, but cannot accurately confirm a certain result.

We agree that this study hypothesizes that nurse density may play a significant role in promoting population health and subsequently population ageing. There is no suggestion of causality throughout any of the paper findings..

The sentence below has been added at the end of the section of “Implications for Practice”.

*However, this study just serves as one of the references for healthcare authorities, and more studies may be required before health authorities consider an increase of density for promoting population health.*

6. Data analysis is somewhat simple. Can we further conduct data analysis and explore at a deeper level.

The nature of the available data is directly related to the depth of analysis that can be performed and the extensive analysis provided herein offers a mature approach to the topic at hand. The methods used have been replicated in top journals with very high impact factors.

We have adopted the following eight data analysis methods in this study for our vigorous data analyses:

1) Scatter plots
2) Pearson’s r
3) Nonparametric
4) Partial correlation
5) Multivariate linear regression (enter)
6) Multivariate linear regression (stepwise)
7) Fisher’s r-to-z transformation
8) Log-transformation

The purposes for including these study methods have been detailed in the manuscript. Our own literature identifies that few other studies have conducted as many analysis methods as ours. We have had two papers using similar approaches to analysis have been published at PlosOne.

1. Large household reduces dementia mortality: A cross-sectional data analysis of 183 populations <https://journals.plos.org/plosone/article?id=10.1371/journal.pone.0263309&utm_source=miragenews&utm_medium=miragenews&utm_campaign=news>

2. Relaxed natural selection contributes to global obesity increase more in males than in females due to more environmental modifications in female body mass <https://journals.plos.org/plosone/article?id=10.1371/journal.pone.0199594>

**References:**

1.	Amiri A, Solankallio-Vahteri T. Nurse staffing and life expectancy at birth and at 65 years old: evidence from 35 OECD countries. International journal of nursing sciences. 2019;6(4):362-70.

2.	You W, Donnelly F. Although in shortage, nursing workforce is still a significant contributor to life expectancy at birth. Public Health Nursing. 2022. doi: <https://doi.org/10.1111/phn.13158>.

3.	You W. Nursing Workforce Protects Against Infant Mortality Globally and Regionally: A Cross-Sectional Data Analysis of 215 Populations. Available at SSRN 4153434. 2022. doi: <https://papers.ssrn.com/sol3/papers.cfm?abstract_id=4153434>.

4.	Amiri A. Role of nurses and midwives in improving patient safety during childbirth: evidence from obstetric trauma in OECD countries. Applied Nursing Research. 2020;56:151343.

5.	Amiri A, Vehviläinen-Julkunen K, Solankallio-Vahteri T, Tuomi S. Impact of nurse staffing on reducing infant, neonatal and perinatal mortality rates: Evidence from panel data analysis in 35 OECD countries. International journal of nursing sciences. 2020;7(2):161-9.

6.	Amiri A, Solankallio-Vahteri T. Analyzing economic feasibility for investing in nursing care: evidence from panel data analysis in 35 OECD countries. International journal of nursing sciences. 2020;7(1):13-20. doi: 10.1016/j.ijnss.2019.06.009.

7.	Amiri A, Solankallio-Vahteri T, Tuomi S. Role of nurses in improving patient safety: evidence from surgical complications in 21 countries. International journal of nursing sciences. 2019;6(3):239-46.

8.	Amiri A, Solankallio-Vahteri T. Nurse-staffing level and quality of acute care services: evidence from cross-national panel data analysis in OECD countries. International journal of nursing sciences. 2019;6(1):6-16.
