# Supplementary material for: Greater nurse density correlates to higher level of population ageing globally, but is more prominent in developed countries
Source: PLoS One. 2023 Sep 29;18(9):e0292371. doi: 10.1371/journal.pone.0292371 (PMC10540962; doi:10.1371/journal.pone.0292371)
Supplement: Attachment — Submitted filename: 0 Response to Review Decision.docx [file pone.0292371.s006.docx]

PONE-D-22-35617R2

Greater nurse density correlates to higher level of population ageing globally, but is more prominent in developed countries.

PLOS ONE

Journal Requirements:

Please review your reference list to ensure that it is complete and correct. If you have cited papers that have been retracted, please include the rationale for doing so in the manuscript text, or remove these references and replace them with relevant current references. Any changes to the reference list should be mentioned in the rebuttal letter that accompanies your revised manuscript. If you need to cite a retracted article, indicate the article’s retracted status in the References list and also include a citation and full reference for the retraction notice.

Authors: We have checked and amended the references. We ensure that:

1) Every reference cited in the text is also present in the reference list (and vice versa)

2) Follow the standard reference style of the PlosOne journal.

3) Each journal articles have the DOI’s if the journals have allocated them to the articles.

4) Webpage reference have the title, hyperlink and access data.

We do not need to cite a retracted or unpublished article in this study.

The details about all the amendments on the references can be viewed in the section of References in the revised manuscript with track changes.

6. Review Comments to the Author

Reviewer #3: The author has made significant revisions to the suggestions mentioned above, and will further refine the language of the article to avoid ambiguity.

Authors: We have tried to refine the language of the article for improving the clarity and readability.

Does the author have any methods, interventions, and subsequent research to address this topic? Does rationality and necessity exist, and is there any significance for further in-depth research. The author can briefly narrate

Authors: Based on the study finding in this article, we suggested that further studies into the long-run elasticity of nurse staffing level on population ageing may be needed. For instance, what percentage of increase of nursing staff would be required to meet every 1% increase of an ageing population. The expected study area has been added at the end of the Conclusion.
